# CO_2_ Activation by Small Copper, Cobalt, and Yttrium Oxide Clusters Probed via Infrared Multiple‐Photon Dissociation Spectroscopy

**DOI:** 10.1002/cphc.70559

**Published:** 2026-09-07

**Authors:** Pavol Mikolaj, Joost M. Bakker, Sandra M. Lang

**Affiliations:** ^1^ Institute of Physical Chemistry II University of Ulm Ulm Germany; ^2^ HFML‐FELIX Nijmegen The Netherlands; ^3^ Institute for Molecules and Materials Radboud University Nijmegen The Netherlands

**Keywords:** carbonate formation, CO_2_ activation, gas‐phase clusters, infrared spectroscopy, metal oxide

## Abstract

The interaction of CO_2_ with Cu, Co, and Y oxide clusters of different size, composition, and charge is investigated via infrared multiple‐photon dissociation (IR‐MPD) spectroscopy. The IR‐MPD spectra of M_*x*_O_*y*_(CO_2_)^+/−^ (M = Cu, Co, Y) complexes reveal the non‐activated binding of CO_2_ to all cationic clusters. In contrast, CO_2_ is activated by all anions via formation of carbonate‐like CO_3_ units. Density functional theory(DFT) calculations on M_2_O_2_^+/−^ model systems suggest that for CO_2_ activation in general, the energy difference between the cluster highest occupied molecular orbital (HOMO) and the CO_2_ lowest unoccupied molecular orbital (LUMO) is crucial. The binding motif of the activated CO_2_ molecule, however, depends on a fine interplay between the nature of the orbitals close to the HOMO and the oxidation state of the metal atoms. CO_3_ formation is favored when the cluster HOMO or any energetically close lying molecular orbital (MO) is at least partially localized on one of the cluster oxygen atoms. In contrast, if such MOs are energetically less favorable, *η* ^2^‐(C,O) and *η* ^2^‐(O,O) binding to one of the metal atoms can be favored, provided the already positively charged metal atom can transfer electron density, i.e., adopt an even higher oxidation state.

## Graphical abstract

Infrared multiple photon dissociation spectroscopy is used to study the activation of CO_2_ on Cu‐, Co‐, and Y‐oxide clusters. While CO_2_ activation is not possible on cationic clusters, the binding motif on anionic clusters depends on a fine interplay between the nature and energetic location of the molecular orbitals and the charge (oxidation state) on the metal atoms.

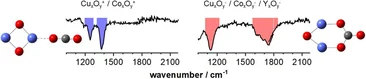

## 1 Introduction

The hydrogenation of carbon dioxide represents one of the promising strategies to decarbonize the earth’s atmosphere while simultaneously synthesizing valuable chemicals and fuels [1, 2, 3]. However, for this process to be economically viable, the development of highly efficient, selective, and durable catalytic materials is required [4, 5]. Currently, the most common industrial catalyst for CO_2_ hydrogenation to methanol is a binary Cu/ZnO‐based material [2, 3, 6]. Although widely used, its large‐scale application is often hampered by deactivation due to sintering and carbon deposits [7].

Mechanistically, mono‐metallic Cu‐based catalysts favor the production of methanol, which is believed to be formed via the formate pathway, with HCOO* as a key reaction intermediate [8, 9, 10, 11, 12]. However, studies on well‐defined model systems showed that the production of other molecules, such as higher alcohols, methane, or formic acid can potentially be achieved by altering the Cu catalyst through, e.g., transition metal alloying [10, 13, 14], the addition of alkali/alkaline earth promotors [10], or the addition of water to the feed [15]. A second well‐known and commonly used catalyst material for CO_2_ hydrogenation is cobalt. However, in contrast to Cu‐based catalysts, Co‐based materials typically promote C─C coupling, thus favoring the formation of longer carbon chains and higher alcohols (C2+ products) [7, 10, 16, 17]. While the exact reaction mechanism remains under debate, it is suggested that both CO* and HCOO* may act as key reaction intermediates [10, 17].

Beyond the nature of the transition metal and the use of promoters, the presence of oxygen can significantly influence the catalytic performance. In particular, Cu─O interfaces [18] and subsurface oxygen [10, 19, 20, 21] have been identified to serve as active sites that facilitate CO_2_ adsorption and conversion in both thermal catalysis and electrocatalysis. Similarly, the synergy between metallic Co and CoO phases has been shown to be beneficial, improving the activity and product selectivity for methanol on a Co/Si_*x*_ catalyst [22] and ethanol on a Co/AlO_*x*_ catalyst [23].

Despite enormous efforts over the last decade to tune and improve the properties of both Cu‐ and Co‐based catalysts for CO_2_ hydrogenation, several fundamental aspects remain incompletely understood. These include the nature of the active sites, the key reaction intermediates, and the exact reaction mechanisms [7, 9]. To address these gaps, it is important not only to study simplified model systems but also to focus on individual reaction steps. The first and thus the most fundamental of these reaction steps involves the adsorption, activation, and potential dissociation of the CO_2_ molecule. Model studies revealed that CO_2_ generally adsorbs rather weakly on flat, stepped, and kinked Cu surfaces [24, 25], whereas it can be activated on cobalt surfaces [26, 27, 28]. Similarly, experimental studies using infrared multiple photon dissociation (IR‐MPD) spectroscopy demonstrated that cationic copper and cobalt clusters cannot activate CO_2_ [29, 30, 31]. While theoretical simulations predicted both non‐activated and activated adsorption on neutral clusters [32, 33, 34, 35, 36], dissociation to CO was found to be kinetically hindered for all Cu clusters. However, further experiments also revealed that cationic transition‐metal‐doped Co_*x*_^+^ as well as anionic Cu^−^, Cu_*x*_O_*y*_^−^, Cu_*x*_C^−^, Co_*x*_^−^, and CuO^−^ are able to activate and in some cases even dissociate CO_2_ [37, 38, 39, 40, 41, 42, 43]. In addition, computational studies showed that the replacement of one Cu atom in a neutral Cu_4_ cluster by cobalt increases the activity of the cluster, leading to the spontaneous bending of the otherwise linear CO_2_ and an energetic stabilization of the formed complex [34].

To gain deeper insight into the adsorption and potential activation of CO_2_ on Cu‐ and Co‐particles and specifically to investigate the role of oxygen in this interaction, we utilize few‐atom clusters with exactly known size, composition, and charge state as model systems and study their CO_2_ complexes with IR‐MPD spectroscopy. In addition to copper oxide and cobalt oxide clusters, we expanded our investigation to include yttrium oxide clusters. The comparison with yttrium is particularly interesting because it has a considerably lower electronegativity than cobalt and copper [44], it is a highly oxophilic element [45], and it readily adopts higher oxidation states. Furthermore, a well‐balanced amount of Y_2_O_3_ has been shown to enhance the catalytic activity of a Cu/ZnO/Al_2_O_3_ catalyst in methanol synthesis by preventing Cu particle aggregation and improving the Cu^2+^ reducibility [46]. Previous experimental and theoretical gas‐phase studies have also indicated that neutral and cationic yttrium atoms can dissociate CO_2_ [47, 48], while YO^+^ activates CO_2_ via CO_3_ formation only after the adoption of multiple molecules [49].

## 2 Methods

### 2.1 Experimental Method

Metal (copper, cobalt, and yttrium) oxide clusters were produced via laser ablation of a rotating metal rod in the presence of an O_2_/He mixture (1% for Cu, 0.25% for Co, and 0.055% for Y) and subsequently reacted with a CO_2_/He mixture (2% for Cu and 10% for Co and Y) in an adjacent flow tube reactor, which was held at room temperature throughout the experiments. The reaction mixture was then expanded into vacuum, forming a molecular beam before entering the intracavity region, where it was irradiated by the IR laser beam of the Free‐Electron Laser for Intra Cavity Experiments (FELICE, 1000–2150 cm^−1^; 10 μs pulse duration; spectral width set to approximately 0.5% full=width at half‐maximum (FWHM) of the central frequency) crossing it at an angle of 35°. A few µs after the interaction with FELICE, all clusters were extracted into a reflectron time‐of‐flight mass spectrometer by a set of pulsed high‐voltage plates and detected with a microchannel plate detector [50, 51].

To correct for long‐term source fluctuations, the experiment was operated at twice the FELICE repetition rate, allowing for the recording of reference mass spectra in between successive FELICE pulses. Whenever FELICE was in resonance with an IR‐active vibrational mode of a given cluster, multiple IR photons were absorbed sequentially, leading to the heating of the complex and finally to its fragmentation. The IR‐MPD spectra shown in this contribution represents the depletion $I(\tilde{\nu })/I_{0}$ at wavenumber $\tilde{\nu }$, where $I(\tilde{\nu })$ and *I*_0_ are the mass peak intensities with and without laser light, respectively. Accordingly, a value of 1 means no fragmentation (depletion), and a value of 0 means 100% depletion (i.e., all clusters fragmented). To reduce the IR fluence with which the complexes are irradiated, the whole instrument can be translated up to 300 mm from the focus position, leading to a 30‐fold reduction in intensity but increased overlap between the laser and molecular beam and thus an increased signal‐to‐noise ratio. To further reduce the IR fluence and increase the spectral resolution, the overlap between laser and molecular beams can be purposely misaligned such that the molecular beam only observes the lower‐intensity part of the laser beam.

### 2.2 Theoretical Method

The theoretical exploration of the metal oxide model clusters and their CO_2_ complexes was performed with the use of density functional theory (DFT) employing the Gaussian 16 software package [52]. The electronic exchange and correlation were described by the unrestricted ωB97XD functional [53] and the wavefunctions with the def2‐TZVPD basis set including diffuse functions [54, 55, 56]. For all model systems, we first optimized the geometries of the bare clusters before placing CO_2_ molecules at different trial positions. All geometries were optimized without any symmetry restrictions. After geometry optimization, the vibrational spectra were calculated in the harmonic approximation to ensure that the structures represent local and global minima. For all systems, different spin states (given as spin multiplicity *M* = 2*S* + 1) and the wavefunction stability were verified. The charge and spin distributions were calculated through natural bond orbital (NBO) analysis [57] implemented in the Gaussian software package. Density of states (DOS) and their projections on certain atoms (pDOS) were evaluated with the software package GaussSum [58], and molecular orbitals were visualized with the software package GaussView [59].

## 3 Results and Discussion

### 3.1 Metal Oxide Formation

**FIGURE 1:**
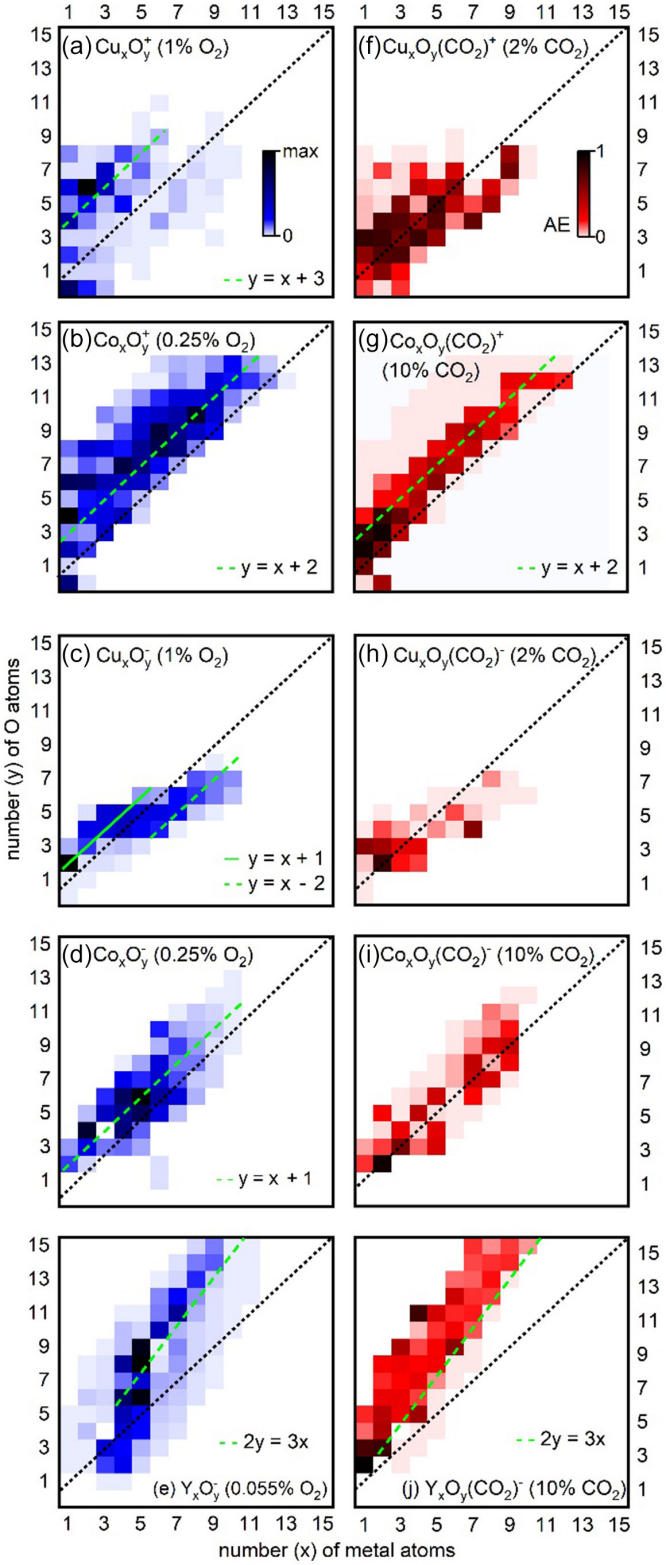
(Left column) Mass spectral intensities of cationic (a) Cu_*x*_O_*y*_^+^ [37] and (b) Co_*x*_O_*y*_^+^ as well as anionic (c) Cu_*x*_O_*y*_^−^ [37], (d) Co_*x*_O_*y*_^−^, and (e) Y_*x*_O_*y*_^−^ clusters produced via laser ablation, in the presence of an oxygen‐enriched carrier gas. Intensities are given in arbitrary units with a linearly scaled color code. (Right column) CO_2_ adsorption efficiency (AE) for the different cluster types calculated with Equation (1). Black dashed lines indicate stoichiometric clusters M_*x*_O_*y*_^+/‍−^ with *x* = *y*, while solid and dashed green lines are drawn to guide the eye to the most abundant (left column) and most reactive (right column) species. Signal intensities increase from light blue/red to dark blue/red.

The left column of Figure 1 displays typical metal oxide cluster distributions. As recently shown [37], laser ablation of a copper target (1% O_2_/He) leads to a wide distribution of cationic Cu_*x*_O_*y*_^+^ clusters (Figure 1a), dominated by highly oxidized species with compositions ranging between *y* = *x* + 1 and *y* = *x* + 4. Cu^+^, CuO_2_^+^, and Cu_2_^+^ are also produced in high intensity. Similarly, the ablation of a cobalt target leads to the production of highly oxidized cationic Co_*x*_O_*y*_^+^ clusters, dominated by species with stoichiometries between *y* = *x* + 1 and *y* = *x* + 4 (Figure 1b). The broadness of the size distribution and the high oxygen content in the cationic Cu_*x*_O_*y*_^+^ and Co_*x*_O_*y*_^+^ clusters suggest the formation of metal–oxide cluster cores, decorated with O_2_ in line with previous hypotheses based on thermal desorption spectroscopy [60], collision‐induced dissociation [61], reactivity studies [62, 63], and photo‐dissociation [64]. It should be noted that cluster distributions formed in a laser vaporization source not only depend on the oxygen content in the carrier gas but also on other source‐specific parameters (such as, O_2_/He pulse timing and duration, laser fluence, size and geometry of the cluster formation region) and can vary strongly depending on the source used. For example, Duncan and coworkers have mainly observed stoichiometric (*x* = *y*) cobalt cations when using a 0.5%–5% O_2_/He mixture [64]. Using a higher oxygen concentration of 10% He, He and coworkers [65] have found oxygen‐rich clusters, in most cases with *y* ≥ *x* + 3, whereas Castleman and coworkers produced highly oxidized mono‐ and di‐cobalt clusters (up to CoO_11_^+^ and Co_2_O_10_^+^) [66].

In contrast, our previously found size distribution of anionic Cu_*x*_O_*y*_^−^ clusters [37] (Figure 1c) is narrower, and the mass spectrum is dominated by slightly oxygen‐rich clusters (*y* = *x* + 1) for *x* ≤ 4 and slightly oxygen‐deficient (*y* = *x *− 1 and *y* = *x −* 2) clusters for *x* ≥ 6, with *x* = 5 representing the transition between both regions. Similarly, the size distribution of anionic Co_*x*_O_*y*_^−^ is narrower (Figure 1d) than the one for cations and is dominated by clusters with a lower oxygen content. In this case, most of the clusters have stoichiometric (*x* = *y*) or slightly oxygen‐rich (*y* = *x* + 1 and *y* = *x* + 2) compositions. This cluster distribution is similar to the ones previously generated by laser ablation in a 10% O_2_/He mixture [66] and of an oxidized Co_3_O_4_ target [67]. Merely ablation of air‐oxidized targets was shown to produce oxygen‐deficient clusters [68, 69].

For yttrium oxide cluster anions Y_*x*_O_*y*_^−^, the size distribution is much broader, and it is generally shifted to more oxygen‐rich species, even in the presence of a very low oxygen concentration of 0.055% (Figure 1e). While clusters containing one and two yttrium atoms are only formed at low intensity, Y_3_O_2_^−^ and Y_3_O_3_^−^ are the preferred tri‐yttrium species. Larger clusters with *x* > 6 are preferably formed with a high oxygen content, which is even slightly higher than the 2*y* = 3*x* stoichiometry of bulk yttrium oxide (Y_2_O_3_). Y_4_O_*y*_^−^ and Y_5_O_*y*_^−^ represent the transition sizes between these two regions, forming species with various oxygen contents ranging from *y* = *x* − 2 to *y* = *x* + 4. The clusters with the highest intensity correspond to (Y_2_O_3_)_3_O^−^, (Y_2_O_3_)_4_O^−^, (Y_2_O_3_)_3_YO_2_^−^, and (Y_2_O_3_)_4_YO_2_^−^, which represent the same stoichiometries as previously found to be particularly stable for cationic species [70, 71]. It should be noted that the oxygen content of these clusters is considerably higher than that of clusters generated by laser ablation of an air‐oxidized target without O_2_ in the carrier gas [72].

The observed overall trend of increasing number of oxygen atoms in the cluster following Cu < Co < Y is consistent with an increasing metal–oxygen binding energy [45, 73]. The most intense cluster sizes of Cu_*x*_O_*y*_^−^ (*x* ≥ 4) and Co_*x*_O_*y*_^−^ (*x* ≥ 3) have O/metal ratios close to the configuration of bulk Cu(II)O and Cu_2_(I)O [74], as well as Co(II)O and Co_3_(II,III)O_4_ [75]. This suggests the coexistence of Cu(I) and Cu(II) as well as Co(II) and Co(III) oxidation states in these oxide clusters. In contrast, smaller clusters (*x* ≤ 4 for Cu and *x* ≤ 3 for Co) are more oxygen‐rich, resulting in larger O/metal ratios. If the preferred Cu and Co oxidation states are also found in these clusters, some of the oxygen atoms must be bound as O_2_ units.

The situation is somewhat different for yttrium oxide: Here, clusters with *x* = 3 are oxygen‐deficient compared to bulk Y_2_O_3,_ while clusters with *x* ≥ 6 are slightly more oxygen‐rich. For the oxygen‐deficient species, this suggests the coexistence of multiple oxidation states or a segregation in oxygen‐free and oxidized parts within the cluster as previously also observed for silicon oxide [76, 77]. For the oxygen‐rich clusters, the formation of O_2_ would also be possible without exceeding the typical +3 oxidation state of yttrium. The formation of such species has, for example, been shown for Y_6_O_10_^+^, but most of the species studied so far contain atomic oxygen [70].

### 3.2 Reactivity of Metal‐Oxide Clusters With CO_2_

The produced metal oxide clusters were subsequently reacted with a CO_2_/He reaction gas (2% for Cu and 10% for Co and Y measurements) in a flow tube reactor. The CO_2_ concentration, gas valve timing with respect to the cluster production, as well as the opening time (determining the pressure in the flow tube reactor), were adjusted for preferred production of M_*x*_O_*y*_(CO_2_), while adsorption of more than one CO_2_ molecule could not be avoided for some species. To compare the reactivity of the different clusters, the right column of Figure 1 shows the CO_2_ adsorption efficiency (AE), illustrated in color map representations and calculated for each cluster size as

(1) $$\mathrm{AE}=\frac{\sum_{z}I\left(M_{x}O_{y}{\left({\mathrm{CO}}_{2}\right)}_{z}\right)}{I\left(M_{x}O_{y}\right)+\sum_{z}I\left(M_{x}O_{y}{\left({\mathrm{CO}}_{2}\right)}_{z}\right)}$$

where *I* denotes the mass peak intensities as extracted from the mass spectrum and *z* = 1,2,… the number of adsorbed CO_2_.

Figure 1f shows that Cu_*x*_O_*y*_(CO_2_)^+^ products are primarily formed for oxygen‐deficient and near‐stoichiometric clusters, while products of highly oxidized clusters (*y* > *x* + 2) are not observed in notable quantities [37]. In the case of Co_*x*_O_*y*_^+^ the highest AE is observed for slightly oxygen‐rich clusters (*y* = *x* + 2, Figure 1g). The lack of reaction products of highly oxidized clusters may be attributed to either the low reactivity of oxygen‐rich clusters toward CO_2_ and/or to an O_2_/CO_2_ substitution reaction (M_*x*_O_*y*_^+^ + CO_2_ → M_*x*_O_y−2_(CO_2_)^+^ + O_2_). Independent of the exact mechanism, it shows that highly oxidized Cu_*x*_O_*y*_(CO_2_)^+^ and Co_*x*_O_*y*_(CO_2_)^+^ complexes are unstable.

It has been previously found [37] that only few anionic copper oxide clusters form products with CO_2_ (Figure 1h). This has been attributed to the narrower size distribution of bare Cu_*x*_O_*y*_^−^ and the fact that only near‐stoichiometric clusters with *x* ≤ 3 and *y* ≤ *x* + 1 are reactive. For Co_*x*_O_*y*_^−^ considerably more products are observed with stoichiometric (*y* = *x*) and near stoichiometric (*y* = *x* − 1, *y* = *x* + 1) clusters being most reactive. The broadest product distribution is, however, observed for anionic yttrium oxide clusters. Following the production of highly oxidized clusters, also Y_*x*_O_*y*_(CO_2_)^−^ with high oxygen content (2*y* = 3*x* up to 2*y* = 3*x* + 3; Figure 1j) is formed.

### 3.3 IR‐MPD Spectra of Cationic CO_2_ Complexes

To gain insight into the bonding motifs of the CO_2_ molecules and their potential activation, we additionally probed the formed M_*x*_O_*y*_(CO_2_)^+/−^ complexes via IR‐MPD spectroscopy. It should be noted that although the CO_2_ adsorption efficiency of some clusters shown in Figure 1 appears to be high (i.e., all or most bare clusters were converted into products), the absolute intensity of the formed product can still be low due to the inefficient production of the corresponding bare cluster. Low intensity of a CO_2_ product might lead to unreliable IR‐MPD spectra with a low signal‐to‐noise ratio and/or contaminations from the fragmentation process of other cluster sizes. Therefore, in the following, we will only present and discuss IR‐MPD spectra with sufficient quality.

**FIGURE 2:**
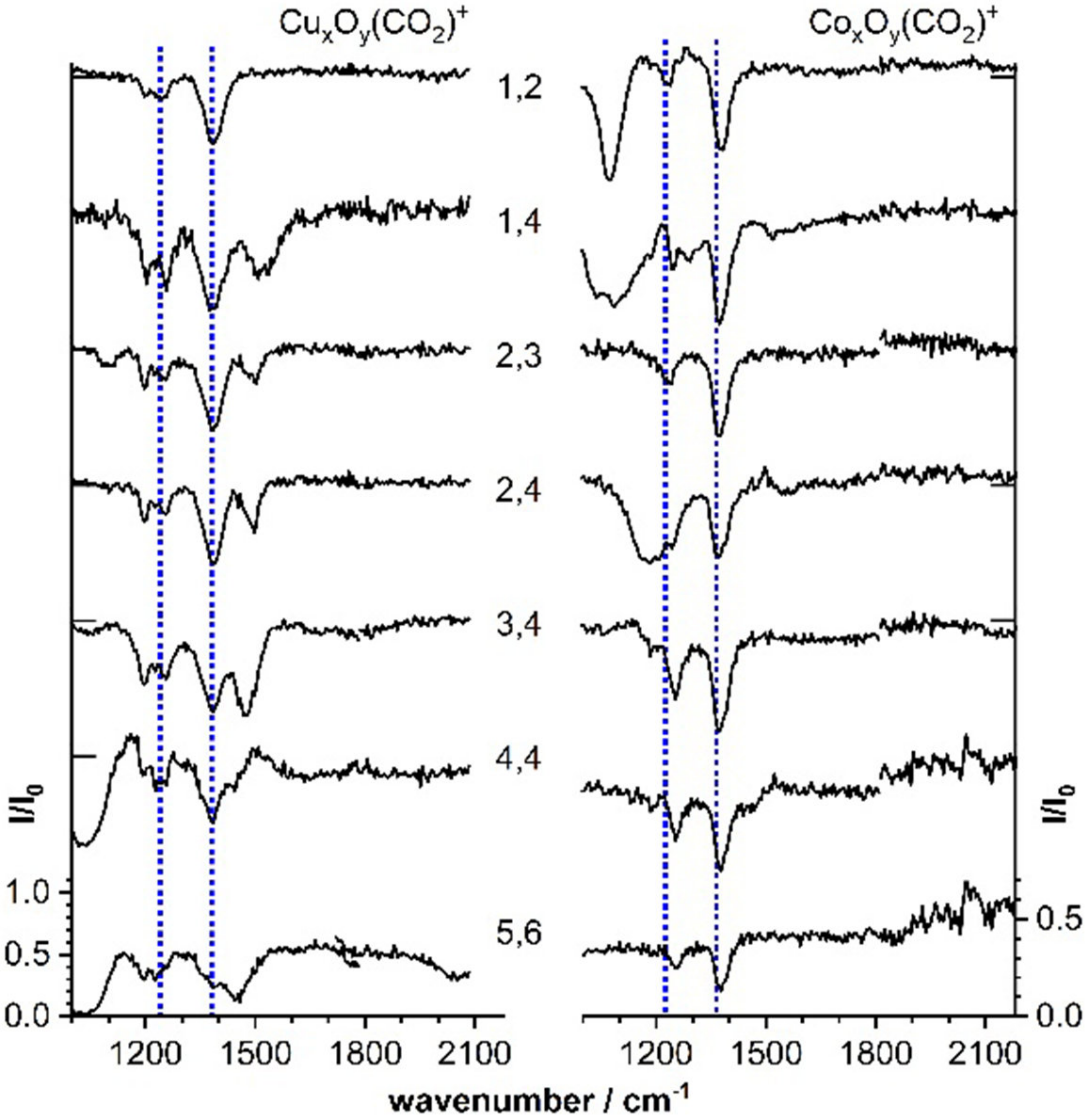
IR‐MPD depletion spectra of cationic (left column) Cu_*x*_O_*y*_(CO_2_)^+^ [37] and (right column) Co_*x*_O_*y*_(CO_2_)^+^ complexes (labeled *x*, *y*). All spectra were measured in two independent runs (1000–1800 and 1800–2180 cm^−1^). Black tick marks on the *y*‐axis indicate zero depletion (*I*/*I*_0_ = 1). The spectra of Cu_5_O_6_(CO_2_)^+^, Co_4_O_4_(CO_2_)^+^ and Co_5_O_6_(CO_2_)^+^ have a strong background and are shifted by 0.4 to increase the visibility of the peaks. The blue dotted lines indicate the Fermi dyad. Spectra of additional Co_*x*_O_*y*_(CO_2_)^+^ complexes are provided in Figure S1.

Figure 2 displays the IR‐MPD spectra of selected cationic Cu_*x*_O_*y*_(CO_2_)^+^ (left column) and Co_*x*_O_*y*_(CO_2_)^+^ (right column) complexes in the spectral region most characteristic of CO_2_. For all Cu_*x*_O_*y*_(CO_2_)^+^ complexes, two peaks centered at 1372–1393 and 1225–1278 cm^−1^ were observed (indicated by a dashed blue line). This doublet has been attributed [37] to the Fermi dyad of linear CO_2_ that arises from the mixing of the symmetric stretching vibration (*ν*_1_) with the first overtone of the bending vibration (2*ν*_2_) [78, 79, 80, 81]. This dyad is IR‐inactive for free CO_2_ but can become IR‐active upon symmetry breaking through adsorption on surfaces [82] or clusters [83, 84]. A similar doublet is also observed in the spectra of the cationic Co_*x*_O_*y*_(CO_2_)^+^ complexes. This indicates that CO_2_ is adsorbed on all Cu_*x*_O_*y*_^+^ and Co_*x*_O_*y*_^+^ clusters as a linear unactivated molecule, independent of the cluster size and metal‐to‐oxygen ratio.

Further bands in the spectra of Cu_*x*_O_*y*_(CO_2_)^+^ have been attributed [37] to weakly bound, non‐activated O_2_ (1400–1550 cm^−1^) [85, 86, 87] as well as the asymmetric CO_2_ stretching mode (*ν*_3_ = 2349 cm^−1^ for free CO_2_) [88], which can become visible at around 1200 cm^−1^ due to the presence of second harmonic radiation of the free electron laser. Unlike Cu_*x*_O_*y*_(CO_2_)^+^, the spectra of Co_*x*_O_*y*_(CO_2_)^+^ do not contain vibrations indicative of O─O stretching of weakly bound O_2_. For CoO_2_(CO_2_)^+^, CoO_4_(CO_2_)^+^, and Co_2_O_4_(CO_2_)^+^, bands between 1070 and 1200 cm^−1^ are observed, which likely arise from a superoxo‐like O_2_ species (this band is also visible in the spectrum of the bare CoO_4_^+^; see Figure S1). Similar bands have previously also been attributed to O─O stretching vibrations in Co_3_O_4_(O_2_)^+^ and Co_4_O_5_(O_2_)^+^ [89]. It should also be mentioned that the spectra of some cluster sizes, such as Cu_5_O_6_(CO_2_)^+^, Co_4_O_4_(CO_2_)^+^, and Co_5_O_6_(CO_2_)^+^, do have a strong background, i.e., the clusters fragment non‐resonantly over the whole investigated spectral region.

### 3.4 IR‐MPD Spectra of Anionic CO_2_ Complexes

**FIGURE 3:**
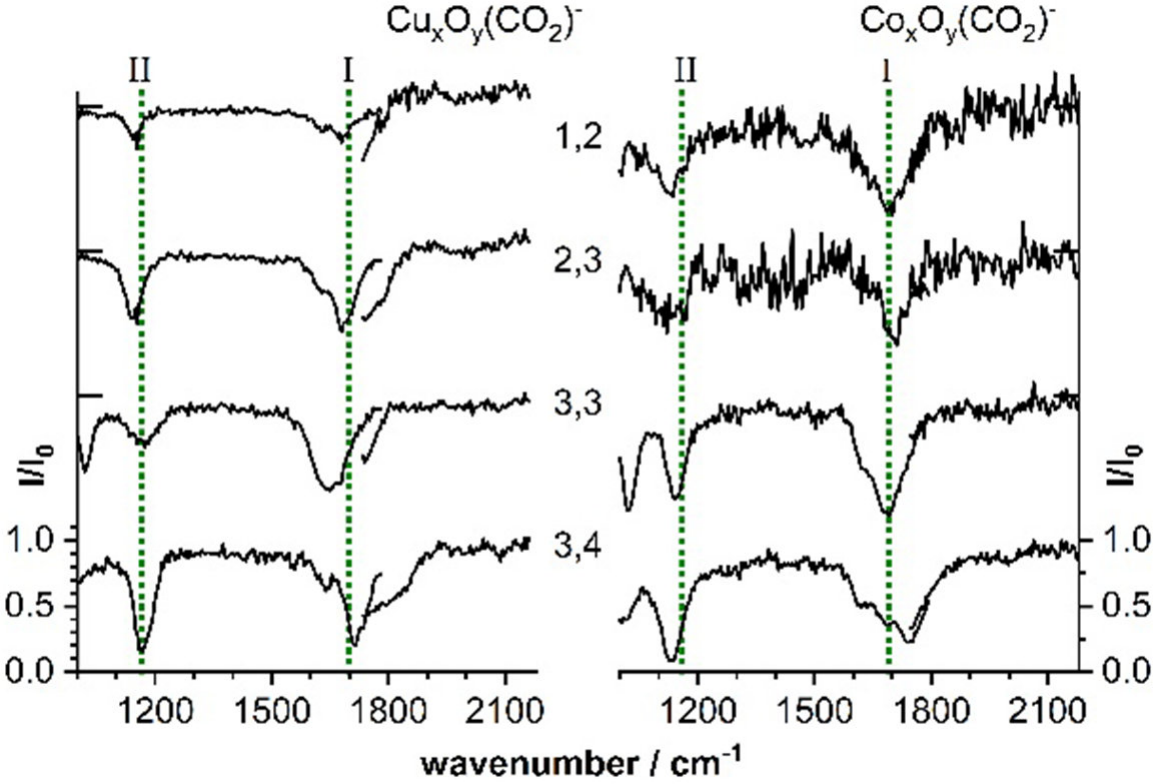
IR‐MPD depletion spectra of anionic (left column) Cu_*x*_O_*y*_(CO_2_)^−^ [37] and (right column) Co_*x*_O_*y*_(CO_2_)^−^ complexes (labeled *x*, *y*). All spectra were measured in two independent runs (1000–1790 and 1735–2150 cm^−1^ for Cu as well as 1000–1780 and 1745–2150 cm^−1^ for Co). The spectra of Cu_*x*_O_*y*_(CO_2_)^−^ in the high wavenumber range were measured with a higher laser fluence than the spectra in the lower wavenumber range. To achieve similar noise level these spectra were scaled by a factor of 0.7. Black tick marks on the *y*‐axis indicate zero depletion (*I*/*I*_0_ = 1). The green dotted lines (bands I and II) are drawn to guide the eye. Spectra of additional Co_*x*_O_*y*_(CO_2_)^−^ complexes are provided in Figure S2.

**FIGURE 4:**
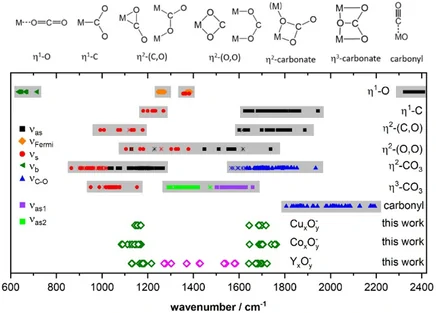
Typical binding motifs of one CO_2_ molecule on atoms and clusters and corresponding vibrational frequencies. The figure is compiled from literature data of various systems experimentally investigated in the gas phase and cold matrixes. Theoretical data (indicated by stars) are only included when insufficient experimental data are available. The figure is adapted from Ref. [90] and modified to include the data for the here investigated Cu_*x*_O_*y*_(CO_2_)^−^, Co_*x*_O_*y*_(CO_2_)^−^, and Y_*x*_O_*y*_(CO_2_)^−^. Open green and magenta data points correspond to bands I/II and III/IV, respectively, defined in Figures 3 and 5. The values are summarized in Tables S1 and S2.

The left column of Figure 3 displays the IR‐MPD spectra of anionic Cu_*x*_O_*y*_(CO_2_)^−^ complexes. Instead of a Fermi dyad as observed for the cationic counterparts, all anionic Cu_*x*_O_*y*_(CO_2_)^−^ display two peaks centered around 1680 and 1150 cm^−1^ (labeled I and II, respectively). By comparing the IR‐MPD spectra of Cu_2_O_4_(CO_2_)^−^ and Cu_3_O_4_(CO_2_)^−^ with DFT calculations [37], we have recently shown that CO_2_ activation results in the formation of CO_3_ bound in a bidentate *η* ^2^‐configuration (see structure *η* ^2^‐carbonate in Figure 4), and bands I and II were assigned to the O─C(O_2_) stretching vibration (*ν*_C─O_) as well as the asymmetric (O)CO_2_ stretching (*ν*_as_) of the CO_3_ unit. In addition to these two characteristic vibrations, a CO_3_ unit also has a symmetric (O)CO_2_ stretching vibration (*ν*_s_), which is at 1063 cm^−1^ for the free carbonate ion [91] and typically in the 850–1150 cm^−1^ range when adsorbed on an atom/cluster [90]. However, it was shown that for Cu_*x*_O_*y*_(CO_2_)^−^, this band can overlap with the O─O stretching motion of a super‐oxo‐like O_2_ unit, making the assignment of this band ambiguous. Therefore, we will not further consider any bands below 1100 cm^−1^.

The assignment to a *η* ^2^‐bound carbonate‐like species is further confirmed by comparison with the literature data shown in Figure 4. This figure represents a compilation of typical CO_2_ vibrations reported for different CO_2_ bonding motifs (details on this figure and corresponding references can be found in Ref. [90]). Comparison of bands I and II with the literature data shows that these bands coincide with the typical vibrations observed for *η* ^2^‐bound CO_3_ units. Based on this comparison, it can also be concluded that this structural motif is present for all the studied Cu_*x*_O_*y*_(CO_2_)^−^ complexes. The viability of CO_2_ binding motif assignment based on such literature data has recently been demonstrated for CO_2_ adsorbed on cationic and anionic magnesium oxide clusters [90].

Analogous to copper oxide, the IR‐MPD spectra of Co_*x*_O_*y*_(CO_2_)^−^ (right column of Figures 3 and S2) also show two bands centered in the regions 1763–1646 and 1166–1086 cm^−1^, diagnostic for *η* ^2^‐bound CO_3_ units. Furthermore, none of the studied copper‐ and cobalt‐based complexes show any bands above1900 cm^−1^ and thus no indications for CO_2_ dissociation into carbon monoxide (see Figure 4 for typical carbonyl vibrations).

**FIGURE 5:**
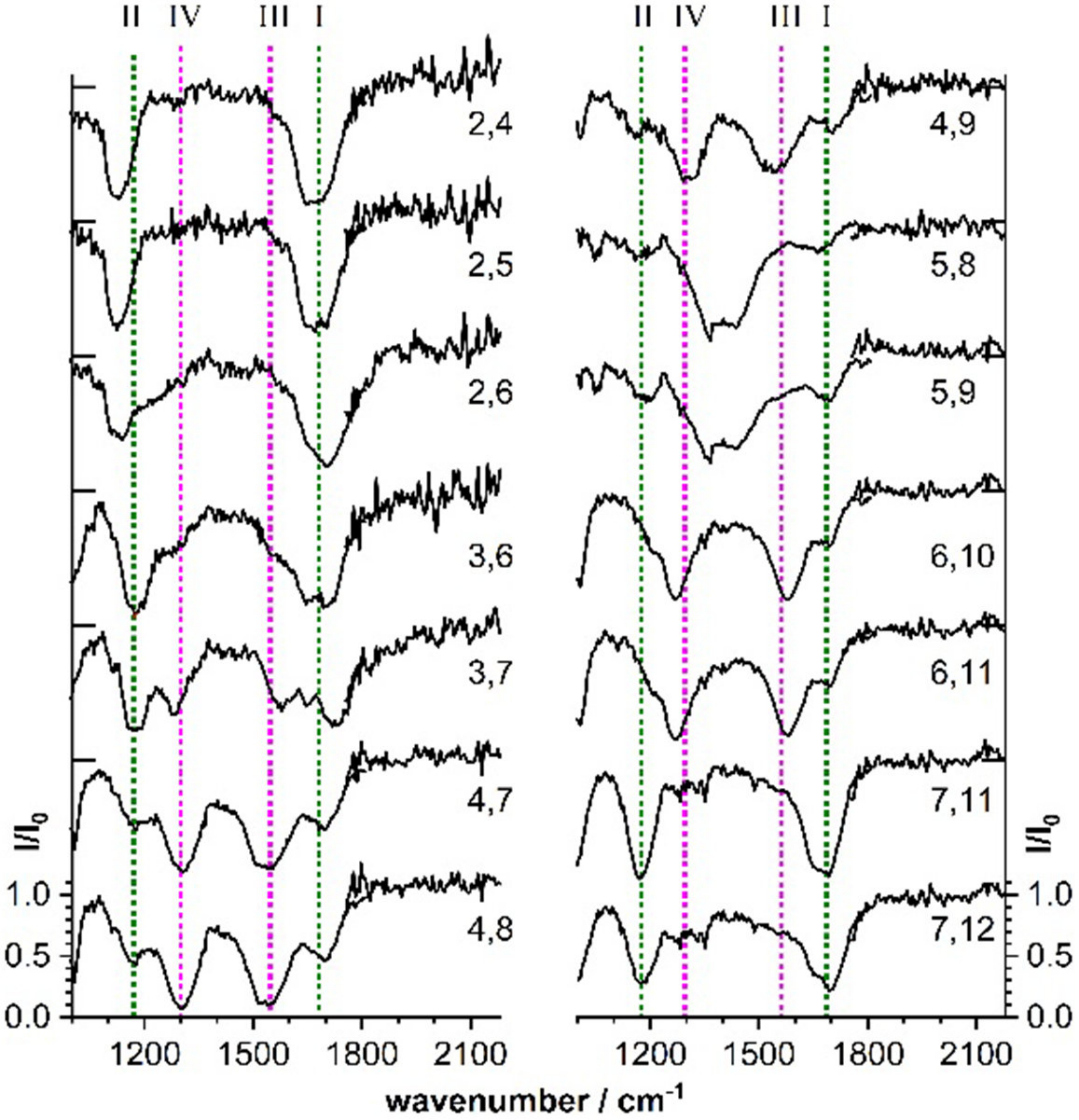
IR‐MPD depletion spectra of anionic Y_*x*_O_*y*_(CO_2_)^−^ complexes (labeled *x*, *y*). All spectra were measured in two independent runs (1000–1805 and 1755–2180 cm^−1^). Black tick marks on the *y*‐axis indicate zero depletion (*I*/*I*_0_ = 1). The green and pink dotted lines are drawn to guide the eye.

Finally, Figure 5 shows the IR‐MPD spectra of Y_*x*_O_*y*_(CO_2_)^−^. The spectra of cluster complexes containing two and seven yttrium atoms (Y_2_O_*y*_(CO_2_)^−^ with *y* = 4–6 and Y_7_O_*y*_(CO_2_)^−^ with *y* = 11, 12) have two bands centered around 1680 cm^−1^/1125 cm^−1^ and 1680 cm^−1^/1170 cm^−1^, respectively (labeled I and II and indicated by dashed green lines) and thus at wavenumbers very similar to those observed for Cu_*x*_O_*y*_(CO_2_)^−^ and Co_*x*_O_*y*_(CO_2_)^−^ (see Figure 4). In contrast, the spectra of complexes containing three yttrium atoms (Y_3_O_*y*_(CO_2_)^−^ with *y* = 6, 7) show two additional bands (labeled III and IV and indicated by dashed pink lines) with a much smaller band splitting. These bands become the dominant ones for Y_4_O_*y*_(CO_2_)^−^ (*y* = 7–9), Y_5_O_*y*_(CO_2_)^−^ (*y* = 8, 9), and Y_6_O_*y*_(CO_2_)^−^ (*y* = 10, 11). Based on Figure 4, bands I and II are again indicative of *η* ^2^‐bound CO_3_ units, whereas bands III and IV are in agreement with *η* ^3^‐bound CO_3_. Due to the lack of a free C─O bond in *η* ^3^‐CO_3_, these bands then correspond to the non‐degenerate asymmetric CO_3_ stretching modes (*ν*_as1_ and *ν*_as2_). The coexistence of both sets of bands for some cluster sizes suggests the presence of two isomeric structures, very similar to the recently reported anionic magnesium oxide clusters [90].

Noteworthy is also the rather large cluster size‐dependent variation of the splitting between bands III and IV, ranging from 309 cm^−1^ for Y_6_O_11_(CO_2_)^−^ to only about 55 cm^−1^ for Y_5_O_8_(CO_2_)^−^. A similar band position and small band splitting as for Y_5_O_8_(CO_2_)^−^ has previously also been reported for Ti_3_O_6_(CO_2_)_2_^−^ and has been explained by a highly symmetric CO_3_ *η* ^3^‐coordinated to three titanium atoms [92]. Similar to copper and cobalt anions, there are no bands observed >1900 cm^−1^ and thus no indications for CO_2_ dissociation into CO.

We conclude that all studied anionic clusters activate CO_2_ via the formation of a carbonate‐like CO_3_ unit without any indications for CO_2_ dissociation. This property is independent of the cluster size, metal‐to‐oxygen ratio, and most importantly, the nature of the metal. For copper and cobalt oxide clusters, the only important parameter is the charge state of the cluster.

### 3.5 DFT Calculations on M_2_O_2_^+/−^ Model Systems

**FIGURE 6:**
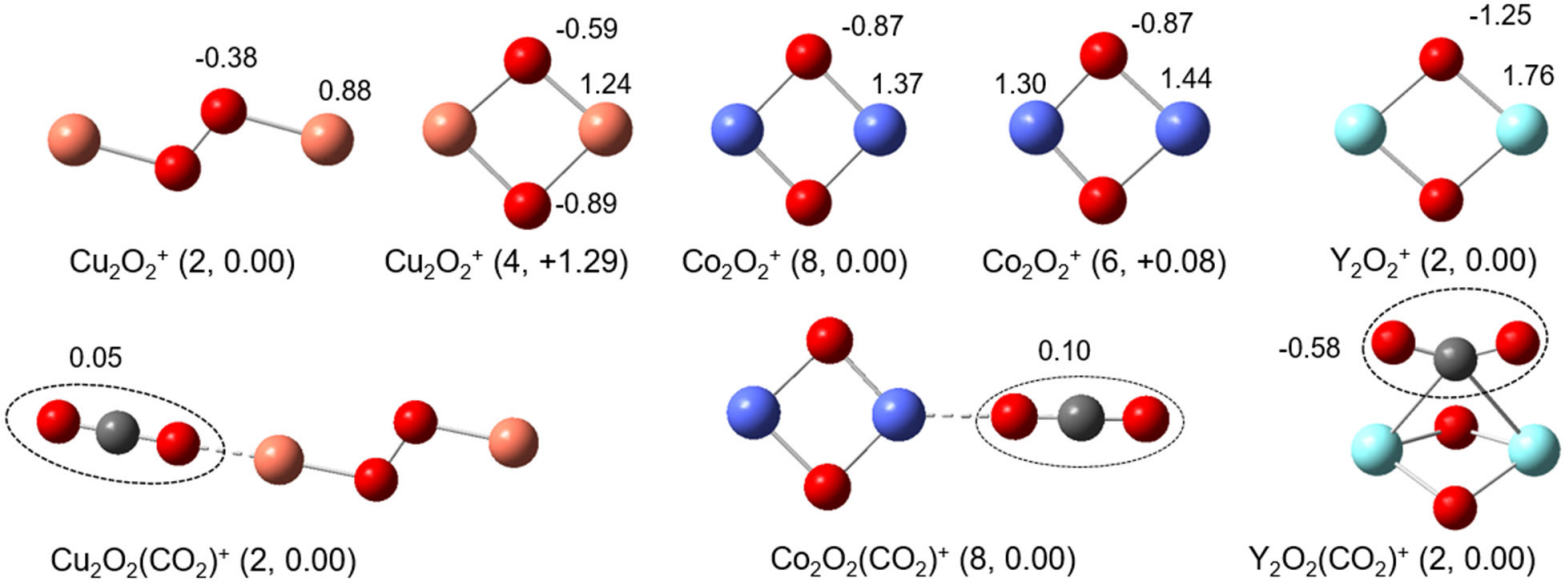
Optimized geometries of cationic M_2_O_2_^+^ (M = Cu, Co, Y) clusters and their CO_2_ complexes. The spin multiplicity and relative energy (in eV) are given in brackets. Local NBO charges (in e) are shown next to the corresponding atoms and the adsorbed CO_2_ molecule, respectively. In case of a symmetric charge distribution only the charge on selected atoms is given. DOS and molecular orbitals of all systems are shown in Figures S3 and S4. Cu, Co, Y, O, and C atoms are depicted by orange, blue, cyan, red, and gray spheres.

To investigate the driving forces for CO_2_ activation and CO_3_ formation, DFT calculations were performed on M_2_O_2_^+/−^ (M = Cu, Y, and Co) serving as a model system. The cationic Y_2_O_2_^+^ and Co_2_O_2_^+^ were both found to adopt a rhombic structure (see Figure 6). In contrast, Cu_2_O_2_^+^ has a bent Cu─O─O─Cu geometry, with the rhombic isomer calculated to be +1.29 eV higher in energy. Due to the electron configuration of yttrium (5s^2^4d^1^) and copper (4s^1^3d^10^), these clusters adopt a low‐spin state with a spin multiplicity of *M* = 2*S* + 1 = 2, in agreement with previous studies [60, 93, 94, 95]. Cobalt (4s^2^3d^7^) oxide clusters, however, exhibit multiple accessible spin states, making the theoretical exploration less straightforward. Our calculations predict the lowest‐energy Co_2_O_2_^+^ isomer to be a rhombus with *M* = 8, though the *M* = 6 (+0.08 eV) isomer is almost isoenergetic. A linear structure (*M* = 8) is significantly higher in energy (+0.59 eV). This contrasts with prior work performed with different basis sets and functionals, as Misaizu and coworkers [61] found a linear Co_2_O_2_^+^ with *M* = 2, while Castleman and coworkers [66] predicted the rhombic structure with *M* = 6 as the lowest‐energy isomer. NBO charge analysis shows that in the rhombic structures, the positive charge on the metal atoms increases in the order Cu (1.24 e)  <  Co (1.37 e)  <  Y (1.76 e), consistent with the decreasing electronegativity of the metals [44]. The charge on the metal in the lowest‐energy Cu_2_O_2_^+^ isomer is even lower (0.88e), which is attributed to the unique binding of an O_2_ unit between the two Cu atoms.

The preferred CO_2_ adsorption mode on Cu_2_O_2_^+^ and Co_2_O_2_^+^ is an *η* ^1^–O coordination, maintaining the linear structure of the CO_2_ molecule. This binding motif is consistent with the observation of the Fermi dyad for these clusters (Figure 2). The cluster‐CO_2_ interaction involves minimal charge transfer (≤0.1 e), and a DOS analysis shows no CO_2_ contribution to the molecular orbitals (MOs) near the HOMO (Figure S4). The absence of any CO_2_ activation can be rationalized by the energetic location of the molecular orbitals of CO_2_ and the DOS of the bare clusters: CO_2_ activation requires electron transfer from the cluster HOMO to the CO_2_ LUMO (2π_u_ orbital) [96]. For Cu_2_O_2_^+^ and Co_2_O_2_^+^, this electron transfer is inhibited by the large energy difference of more than 14 eV between these orbitals (see the DOS in Figure S3).

The situation is different for Y_2_O_2_^+^. For this cluster, the HOMO is at considerably higher energies (at −9.14 eV compared to −13.20 eV for Cu_2_O_2_^−^ and −14.99 eV for Co_2_O_2_^−^), which appears to enable the necessary cluster‐to‐CO_2_ charge transfer. As a consequence, a *η* ^1^–C structure is found as the lowest‐energy isomer, which is 0.36 eV more stable than the *η* ^1^–O isomer. The formation of the *η* ^1^–C structure is accompanied by a charge transfer of 0.58 e from the cluster to the CO_2_ molecule and the (at least partial) location of the HOMO on CO_2_ (Figure S4).

Prior work [49] on YO(CO_2_)_*n*_^+^ (*n* = 2–11) showed the non‐activated adsorption of the first three CO_2_ molecules and CO_3_ formation after the adsorption of a fourth molecule. This activation was attributed to the sum of small consecutive charge donations from each CO_2_ molecule to the clusters, which led to an increased electron density on both the Y and O atoms. The increased negative charge on the O atom was proposed to elevate the O lone‐pair orbital energy, facilitating the overlap with the CO_2_ LUMO and promoting a nucleophilic attack on the C center. Since the charge transfer in these systems is rather small, the effect should be much more pronounced in negatively charged clusters. Therefore, we also studied the anionic M_2_O_2_^−^ clusters (M = Cu, Y, and Co) to test this hypothesis.

**FIGURE 7:**
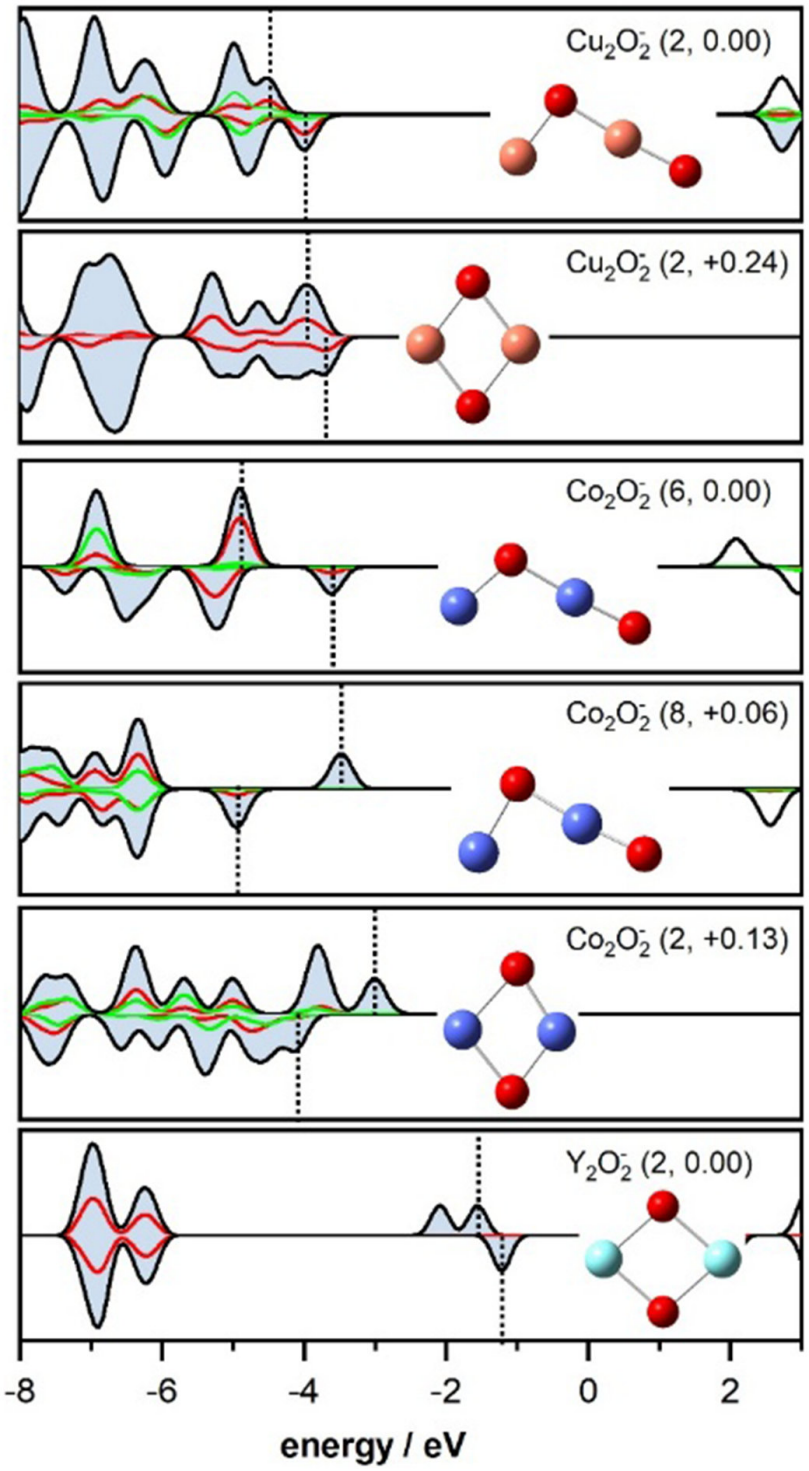
Optimized geometries of anionic M_2_O_2_^−^ (M = Cu, Co, Y) clusters together with their density of states. The total DOS of α‐ (upper panels) and β‐spin (lower panels) electrons are shown in black and the DOS projected on the oxygen atom that participates in CO_3_ formation and the second O atom are shown in red and green, respectively. Gray shaded areas represent occupied states and the HOMOs are indicated by dashed lines. The spin multiplicity and relative energy (in eV) are given in brackets. Cu, Co, Y, and O C atoms are depicted by orange, blue, cyan, and red spheres. Charge and spin distributions are given in Figures S5 and S6.

The lowest energy isomer for Cu_2_O_2_^−^ (*M* = 2) and Co_2_O_2_^−^ (*M* = 6) is a bent M─O─M─O structure (Figures 7 and S5). For both clusters, the rhombic isomers lie relatively close in energy (Cu_2_O_2_^−^ (*M* = 2) rhombus + 0.24 eV; Co_2_O_2_^−^ (*M* = 2) rhombus +0.13 eV). Interestingly, the rhombic isomer of Co_2_O_2_^−^ only has a formal multiplicity of 2 since the two Co atoms are antiferromagnetically coupled (Figure S6). In contrast to the two other metals, Y_2_O_2_^−^ prefers a rhombic structure. Our Cu_2_O_2_^−^ structure is consistent with previous theoretical studies [93]. The bent M─O─M─O structure of Co_2_O_2_^−^ is similar to the previously predicted geometries of neutral [97] Co_2_O_2_ as well as anionic [66] Co_2_O_3_^−^. However, it contrasts with the findings of Castleman and coworkers [66], who predicted the rhombic structure (*M* = 2) as the lowest energy isomer. The addition of the two electrons also profoundly affects the charge distribution (Figure S5): in the copper and cobalt oxides, the positive charge on the metal atoms decrease and the negative charge on the O atoms increases, while in the yttrium oxide the additional negative charge localizes primarily on the Y atoms. Most importantly, the anionic clusters show a considerable energetic upshift of the HOMO (see the gray shaded areas of DOS in Figure 7) compared to the cationic counterparts. This elevation is expected to significantly facilitate electron transfer to the CO_2_ LUMO, thus enhancing CO_2_ activation.

**FIGURE 8:**
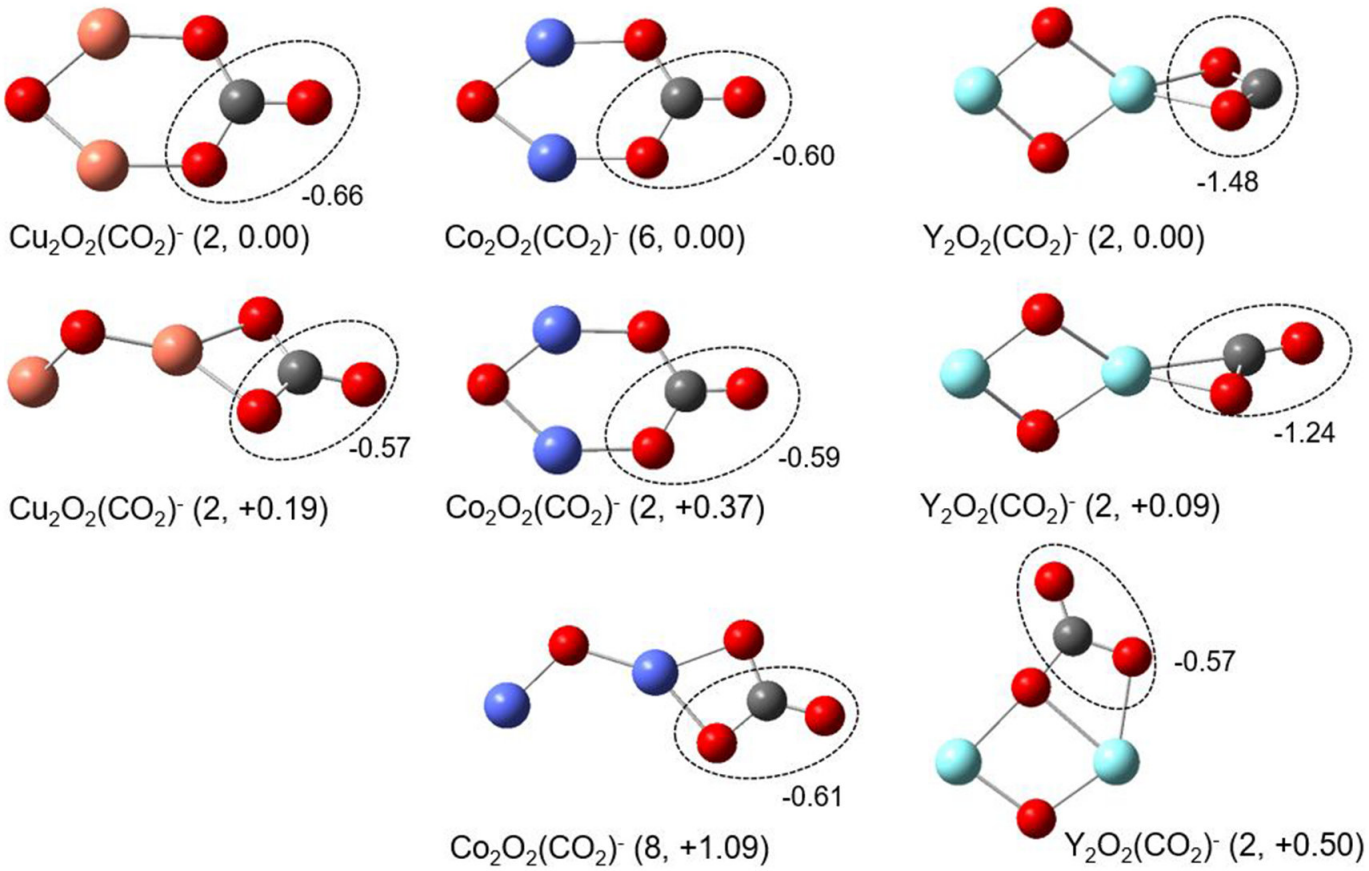
Optimized geometries of several isomeric structures of the anionic M_2_O_2_(CO_2_)^−^ (M = Cu, Co, Y). The spin multiplicity and relative energy (in eV) are given in brackets, local NBO charges (in e) on the adsorbed CO_2_ molecule (indicated by a dashed circle) next to the structure. DOS and molecular orbitals of all systems are shown in Figure S8. Cu, Co, Y, O, and C atoms are depicted by orange, blue, cyan, red, and gray spheres.

Our calculations confirm the experimental finding that for all studied anionic clusters, complexes with activated CO_2_ are the energetically most stable ones (Figure 8). This activation is driven by a considerable charge transfer (>0.5 e) from the cluster to CO_2_ and a significant mixing of the cluster and CO_2_ molecular orbitals (see Figure S8). For Cu and Co oxides, *η* ^2^‐CO_3_ formation is preferred. The calculated frequencies for *ν*_C─O_ and *ν*_as_ of the CO_3_ unit bound to the lowest‐energy isomers are 1679 cm^−1^/1146 cm^−1^ for Cu_2_O_2_(CO_2_)^−^ and 1712 cm^−1^/1197 cm^−1^ for Co_2_O_2_(CO_2_)^−^, in agreement with the IR‐MPD spectra of Cu_*x*_O_*y*_(CO_2_)^−^ and Co_*x*_O_*y*_(CO_2_)^−^. However, for Cu_2_O_2_(CO_2_)^−^, the CO_3_ formation on the rhombic Cu_2_O_2_^−^ isomer is more favorable than on the bent Cu─O─Cu─O isomer (compare a binding energy of 2.52 eV for the rhombic structure with 2.08 eV for the linear structure). For Co_2_O_2_^−^, the lowest‐energy isomer (bent Co─O─Co─O with *M* ‍= ‍6) undergoes a structural rearrangement upon CO_3_ formation, yielding a ring‐like structure (compare structures in Figures ‍7 and ‍8). In contrast, the second Cu_2_O_2_^−^ (*M* = 8, +0.06) retains its bent M─O─M─O structure upon CO_3_ formation but is +1.09 eV less stable. Also, the rhombic isomer (*M* = 2, +0.13 ‍eV) is somewhat destabilized upon CO_3_ formation (+0.37 eV).

This energetic ordering can be explained by inspecting the DOS of the bare clusters (Figure 7). For both Cu_2_O_2_^−^ isomers, the HOMO is (at least partially) localized on the oxygen atom that participates in the CO_3_ formation (indicated by the red curves; see also Figure S7). In contrast, the HOMO (β‐HOMO) of the lowest energy isomer of Co_2_O_2_^−^ (*M* = 6, 0.00) is mainly localized on one of the Co atoms. However, the α‐HOMO is mainly localized on the O atom. In the case of the second isomer (*M* = 8, +0.06), both MOs are localized on the Co atoms, while MOs with noteworthy O contribution are lower in energy. The corresponding Co_*x*_O_*y*_(CO_2_)^−^ is considerably less stable (+1.09 eV). Thus, the key driving force for CO_3_ formation appears to be the (at least partial) localization of the cluster’s HOMO or another energetically close‐lying MO on the O atom that participates in the CO_3_ formation.

This effect is even more pronounced for Y_2_O_2_(CO_2_)^−^. The structural search reveals that CO_3_ formation is not preferred on Y_2_O_2_^−^ but instead a bent CO_2_ unit bound in a *η* ^2^‐(O,O) and *η* ^2^‐(C,O) (+0.09 eV) configuration, respectively, is favored. For Y_2_O_2_^−^, the HOMO (and also several other energetically close‐lying MOs) is localized on the Y atoms, and the O‐localized MOs are about 4 eV lower in energy, hampering CO_3_ formation. However, due to the low electronegativity of Y and its capacity to adopt higher oxidation states, CO_2_ is instead activated by almost exclusive charge transfer from the Y atom, leading to *η* ^2^‐(O,O) and *η* ^2^‐(C,O) binding motifs (NBO charges are given in Figure S9). These binding motifs are found to be highly unstable for the Co and Cu clusters.

The formation of an activated CO_2_ molecule on Y_2_O_2_^−^, however, conflicts with our tentative assignment of the bands in the IR‐MPD spectra to *η* ^2^‐ and *η* ^3^‐CO_3,_ and the calculated frequencies *ν*_s_ = 1151 and *ν*_as_ = 1079 cm^−1^ for this structure also disagree with the IR‐MPD spectra. In contrast, the higher‐energy isomer containing a CO_3_ unit exhibits frequencies (*ν*_C─O_ = 1803 cm^−1^ and *ν*_as_ = 1220 cm^−1^) that are in the experimentally observed spectral range. To determine whether *η* ^2^‐(C,O) and *η* ^2^‐(O,O) binding is unique to the Y_2_O_2_^−^ model system or a general motif, we studied some larger, more oxygen‐rich clusters that better match the experimentally studied stoichiometries: Y_2_O_3_^−^, (Y_2_O_3_)_*n*_O^−^ (*n* = 1–‍3), and (Y_2_O_3_)_*n*_YO_2_^−^ (*n* = 2, 3). Due to their rather large size, we have not performed a full structural search but instead re‐optimized structures previously reported for cationic species [70]. The DOS of Y_2_O_3_^−^, Y_4_O_7_^−^, and Y_5_O_8_^−^ are generally similar to Y_2_O_2_^−^, exhibiting no O contribution to the HOMO (Figure S10). For Y_6_O_10_^−^ and Y_7_O_11_^−^, the HOMO has some contribution of O atoms, but the electron density is distributed over several O atoms and not localized on one specific atom. The only exception is Y_2_O_4_^−^, for which the HOMO is mainly localized on one of the terminal O atoms. Generally, MOs with higher O contribution (which could be involved in CO_3_ formation) are energetically somewhat higher in energy than those for Y_2_O_2_^−^. However, the major difference is the charge distribution: due to the increased cluster size (which leads to the distribution of the net negative charge over many atoms) and the higher oxygen content, the Y atoms already carry a substantial positive charge, typically between 1.20 and 2.01e (the only exception is Y_2_O_3_^−^; see Figure S11).

This high positive charge destabilizes the *η* ^2^‐(C,O) and *η* ^2^‐(O,O) configurations because electron transfer from the already highly positive metal to the CO_2_ molecule seems unfavorable. Indeed, calculations of Y_2_O_3_(CO_2_)^−^, Y_2_O_4_(CO_2_)^−^, and Y_4_O_7_(CO_2_)^−^ confirm the *η* ^2^‐CO_3_ structure as the most stable one (Figure S12). Therefore, while the coexistence of *η* ^2^‐(C,O) and *η* ^2^‐(O,O) isomers in the molecular beam cannot be entirely ruled out, the IR‐MPD bands most likely correspond to *η* ^2^‐CO_3_ and *η* ^3^‐CO_3,_ as suggested above.

Based on these results, we conclude that the energy difference between the cluster HOMO and the CO_2_ LUMO is crucial for favorable charge transfer and CO_2_ activation in general. CO_3_ formation is favored when the cluster HOMO or any MO energetically close to it is at least partially localized on one of the cluster oxygen atoms. Conversely, if such MOs are too low in energy, *η* ^2^‐(C,O) and *η* ^2^‐(O,O) binding to one of the metal atoms can be favored, provided the already positively charged metal atom can transfer electronic charge, i.e., adopt an even higher oxidation state.

## 4 Summary and Conclusion

Laser ablation of Cu, Co, and Y metal targets in the presence of an O_2_/He gas mixture produced oxygen‐rich cationic Cu_*x*_O_*y*_^+^ and Co_*x*_O_*y*_^+^, with oxygen content reaching *y* = *x* + 4. Anionic clusters, however, were less oxygen‐rich, following a trend consistent with the increasing bulk oxidation state: Cu < Co < Y. Subsequent reaction of these clusters with CO_2_ in a flow tube resulted in strongly charge‐state and metal‐dependent product distributions.

IR‐MPD spectroscopy of the formed CO_2_ complexes revealed that all cationic Cu_*x*_O_*y*_^+^ and Co_*x*_O_*y*_^+^ clusters adsorb CO_2_ in a linear, non‐activated fashion. This lack of CO_2_ activation is attributed to a large energy difference between the cluster HOMO and the CO_2_ LUMO, which prevents efficient charge transfer necessary for CO_2_ activation. In stark contrast, all anionic clusters were found to be capable of activating CO_2_ by forming carbonate‐like CO_3_ units. This CO_3_ is preferably bound in a *η* ^2^‐configuration to Cu_*x*_O_*y*_^−^ and Co_*x*_O_*y*_^−^, whereas both *η* ^2^‐ and *η* ^3^‐bound species were observed for Y_*x*_O_*y*_^−^. The activation of the CO_2_ molecule seems to be enabled through a considerable energetic lifting of the cluster HOMO, which facilitates efficient charge transfer. The specific binding motif of the activated CO_2_ molecule appears to depend on a fine interplay between the nature and energetic location of the molecular orbitals and the charge (oxidation state) of the metal atoms. CO_3_ formation is preferred when the cluster HOMO (or any energetically close‐lying MO) is at least partially localized on one of the oxygen atoms. If these MOs are energetically less favorable, *η* ^2^‐(C,O) and *η* ^2^‐(O,O) binding to one of the metal atoms can be favored, provided the already positively charged metal atom can donate electronic density and thus adopt an even higher oxidation state.

This combined experimental and theoretical study provides fundamental insight into the main driving forces for CO_2_ activation and the resulting binding motifs on metal oxide clusters. Future work will concentrate on validating these findings across a wider range of metal oxides, particularly those containing metal atoms that can readily adopt high oxidation states.

## Funding

This study was supported by the Horizon 2020 Framework Programme (955650 and 654148), Deutsche Forschungsgemeinschaft (INST 40/575‐1 FUGG), and Ministerium für Wissenschaft, Forschung und Kunst Baden‐Württemberg.

## Conflicts of Interest

The authors declare no conflicts of interest.

## Supporting information

The supporting information include additional IR‐MPD spectra as well as geometric and electronic details of the calculated structures.

## Data Availability

The data that support the findings of this study are available on request from the corresponding author. The data are not publicly available due to privacy or ethical restrictions.
