# Supplementary material for: CO2 Activation by Small Copper, Cobalt, and Yttrium Oxide Clusters Probed via Infrared Multiple‐Photon Dissociation Spectroscopy
Source: Chemphyschem. 2026 Sep 7;27(17):e70559. doi: 10.1002/cphc.70559 (PMC13549768; doi:10.1002/cphc.70559)
Supplement: Supplementary file 1 [file CPHC-27-e70559-s001.pdf]

Supporting information to

CO_2 Activation by Small Copper, Cobalt, and Yttrium Oxide Clusters Probed via IR-MPD Spectroscopy

Pavol Mikolaj, Joost M. Bakker, and Sandra M. Lang

S1 Supplementary IR-MPD spectra

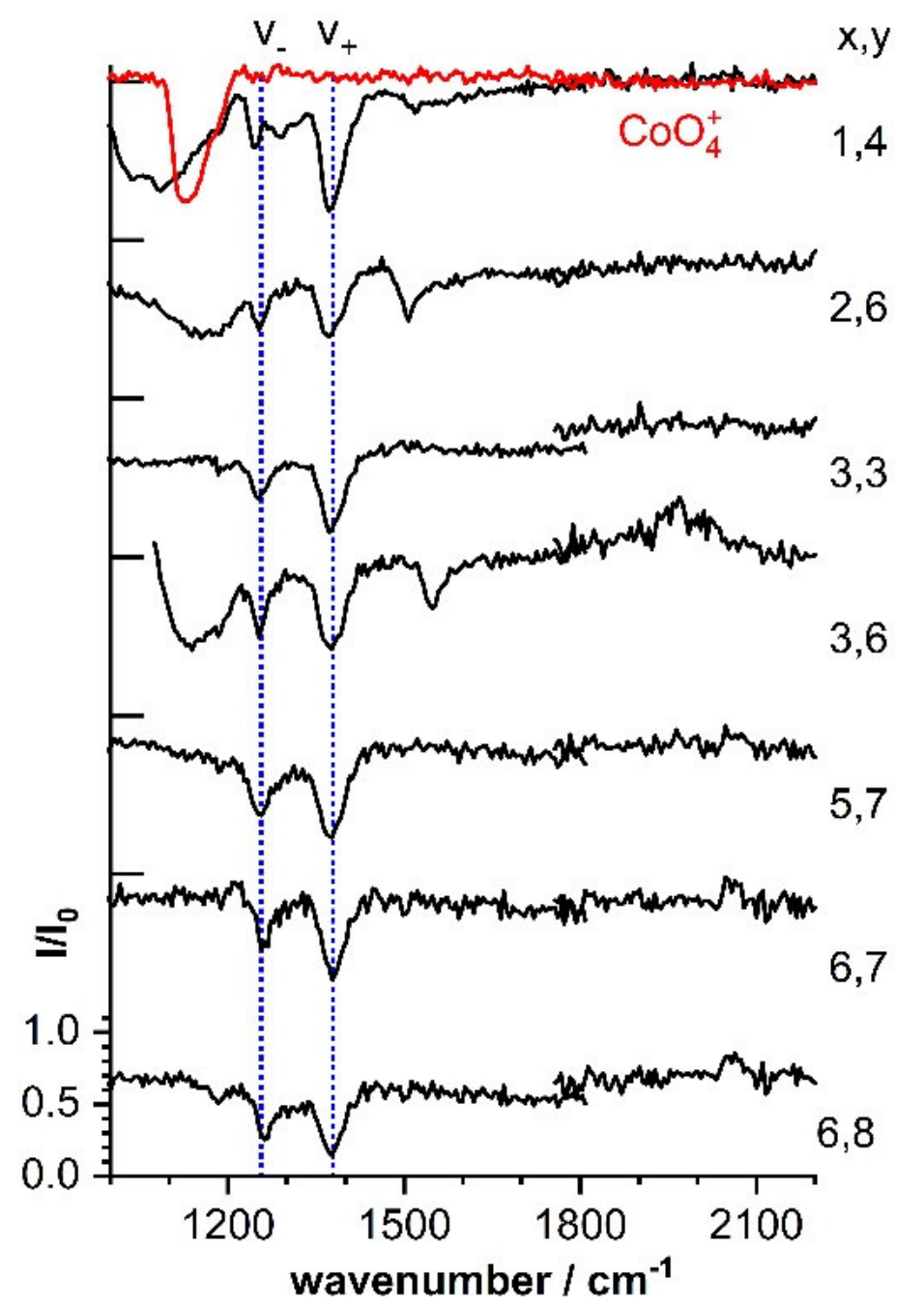

Figure S1: IR-MPD spectra (in black) of cationic $Co_xO_y(CO_2)^+$ complexes not shown in Figure 2 of the main text. In addition, the spectrum of the bare CoO_4^+ clusters (in red) is shown. Blue dotted lines indicate the Fermi dyad. Black tick marks on the y-axis indicate zero depletion ($I/I_0 = 1$).

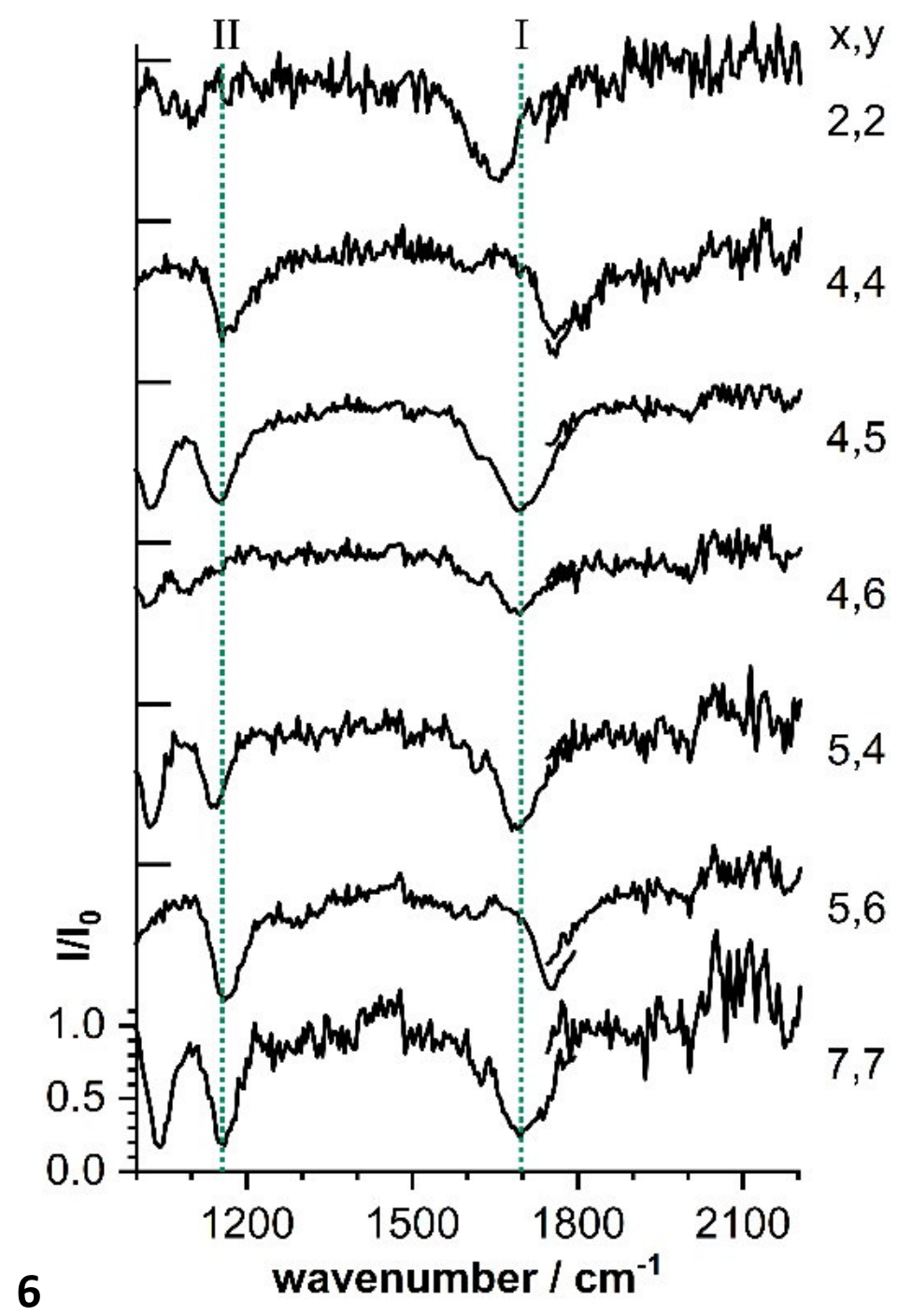

Figure S2: IR-MPD spectra of anionic $Co_xO_y(CO_2)^-$ complexes not shown in Figure 3 of the main text. The green dotted lines indicate modes of the formed CO_3 unit, discussed in Section 3.4. Black tick marks on the y-axis indicate zero depletion ($I/I_0 = 1$).

Table S1. Experimental band positions (in cm⁻¹; estimated error of ±5 cm⁻¹) for cationic $Cu_xO_y(CO_2)^+$ and $Co_xO_y(CO_2)^+$. Bold text denotes clusters discussed in the main text; further $Cu_xO_y(CO_2)^+$ data are reported in ref. [1] and additional $Co_xO_y(CO_2)^+$ cluster data is provided in Figure S1.

Cluster	ν_-	ν_+
**$CuO_2(CO_2)^+$**	1240	1386
$CuO_3(CO_2)^+$	1243	1355
**$CuO_4(CO_2)^+$**	1257	1384
**$Cu_2O_3(CO_2)^+$**	1250	1384
**$Cu_2O_4(CO_2)^+$**	1251	1385
**$Cu_3O_4(CO_2)^+$**	1253	1376
$Cu_3O_5(CO_2)^+$	1246	1389
$Cu_4O_3(CO_2)^+$	1227	1382
**$Cu_4O_4(CO_2)^+$**	1236	1382
$Cu_5O_5(CO_2)^+$	1392	1441
**$Cu_5O_6(CO_2)^+$**	1389	1449
$Cu_6O_4(CO_2)^+$	1264	1374

Cluster	ν_-	ν_+
**$CoO_2(CO_2)^+$**	1229	1379
**$CoO_4(CO_2)^+$**	1248	1377
**$Co_2O_3(CO_2)^+$**	1232	1375
**$Co_2O_4(CO_2)^+$**	1239	1375
$Co_2O_6(CO_2)^+$	1253	1375
$Co_3O_3(CO_2)^+$	1254	1377
**$Co_3O_4(CO_2)^+$**	1253	1376
$Co_3O_6(CO_2)^+$	1253	1376
**$Co_4O_4(CO_2)^+$**	1253	1377
**$Co_5O_6(CO_2)^+$**	1254	1378
$Co_5O_7(CO_2)^+$	1255	1375
$Co_6O_7(CO_2)^+$	1262	1379
$Co_6O_8(CO_2)^+$	1263	1376

Table S2. Experimental band positions (cm⁻¹; estimated error of ±5 cm⁻¹) for anionic $Y_xO_y(CO_2)^-$, $Co_xO_y(CO_2)^-$, and $Cu_xO_y(CO_2)^-$. Bold text denotes clusters discussed in the main text; further $Cu_xO_y(CO_2)$ data are reported in ref. [1] and additional $Co_xO_y(CO_2)^+$ cluster data is provided in Figure S2.

Cluster	II	IV	III	I
**$Y_2O_4(CO_2)^-$**	1129			1672
**$Y_2O_5(CO_2)^-$**	1129			1677
**$Y_2O_6(CO_2)^-$**	1132			1698
**$Y_3O_6(CO_2)^-$**	1179			1702
**$Y_3O_7(CO_2)^-$**	1177	1278	1584	1722
**$Y_4O_7(CO_2)^-$**	1182	1303	1542	1683
**$Y_4O_8(CO_2)^-$**	1182	1303	1541	1685
**$Y_4O_9(CO_2)^-$**	1165	1303	1534	1695
**$Y_5O_8(CO_2)^-$**	1178	1372	1426	1643
**$Y_5O_9(CO_2)^-$**	1186	1370	1429	1678
**$Y_6O_{10}(CO_2)^-$**	1216	1272	1579	1685
**$Y_6O_{11}(CO_2)^-$**	1131	1272	1581	1682
**$Y_7O_{11}(CO_2)^-$**	1174			1673
**$Y_7O_{12}(CO_2)^-$**	1180			1695

Cluster	II	I
**$CuO_2(CO_2)^-$**	1159	1691
$CuO_3(CO_2)^-$	1150	1700
**$Cu_2O_3(CO_2)^-$**	1145	1685
$Cu_2O_4(CO_2)^-$	1155	1687
**$Cu_3O_3(CO_2)^-$**	1168	1645
**$Cu_3O_4(CO_2)^-$**	1170	1717

Cluster	II	I
**$CoO_2(CO_2)^-$**	1122	1689
$Co_2O_2(CO_2)^-$	1086	1646
**$Co_2O_3(CO_2)^-$**	1112	1705
**$Co_3O_3(CO_2)^-$**	1143	1687
**$Co_3O_4(CO_2)^-$**	1132	1742
$Co_4O_4(CO_2)^-$	1171	1763
$Co_4O_5(CO_2)^-$	1149	1699
$Co_4O_6(CO_2)^-$	1090	1694
$Co_5O_4(CO_2)^-$	1143	1695
$Co_5O_6(CO_2)^-$	1166	1757
$Co_7O_7(CO_2)^-$	1158	1703

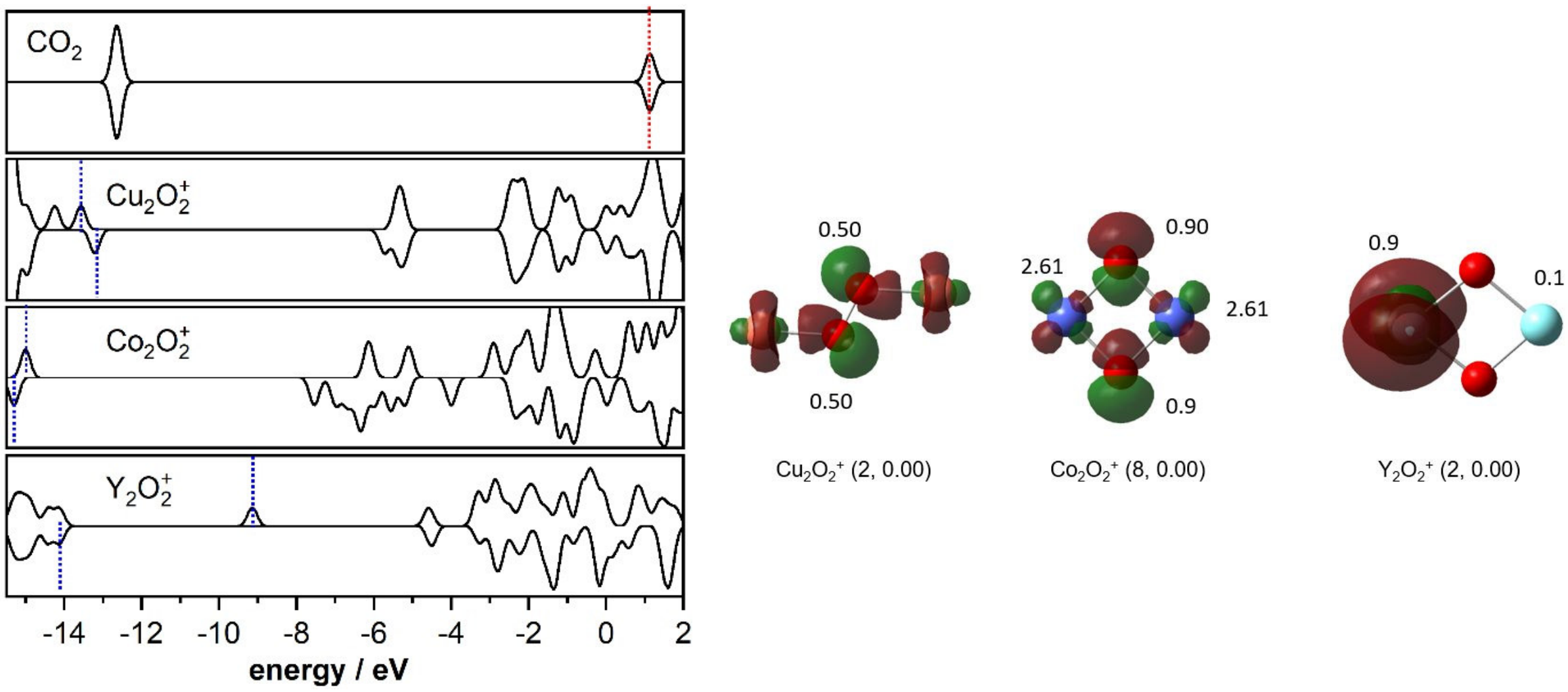

Figure S3: Total density of states (DOS) of the calculated lowest energy structures of CO_2, $Cu_2O_2^+$, $Co_2O_2^+$, and $Y_2O_2^+$ shown in Figure 6 of the main text. Shown are the DOS of α-spin (upper panels) and β-spin (lower panels) electrons. The dashed red line indicates the LUMO of CO_2 molecule and the dashed blue lines indicate the α-spin and β-spin HOMOs of the clusters. Also shown are the corresponding optimized geometries together with the isosurfaces (isovalue 0.05) of the HOMO. The numbers denote the major components of the excess spin. Cu, Co, Y and O atoms are depicted by orange, blue, cyan, and red spheres.

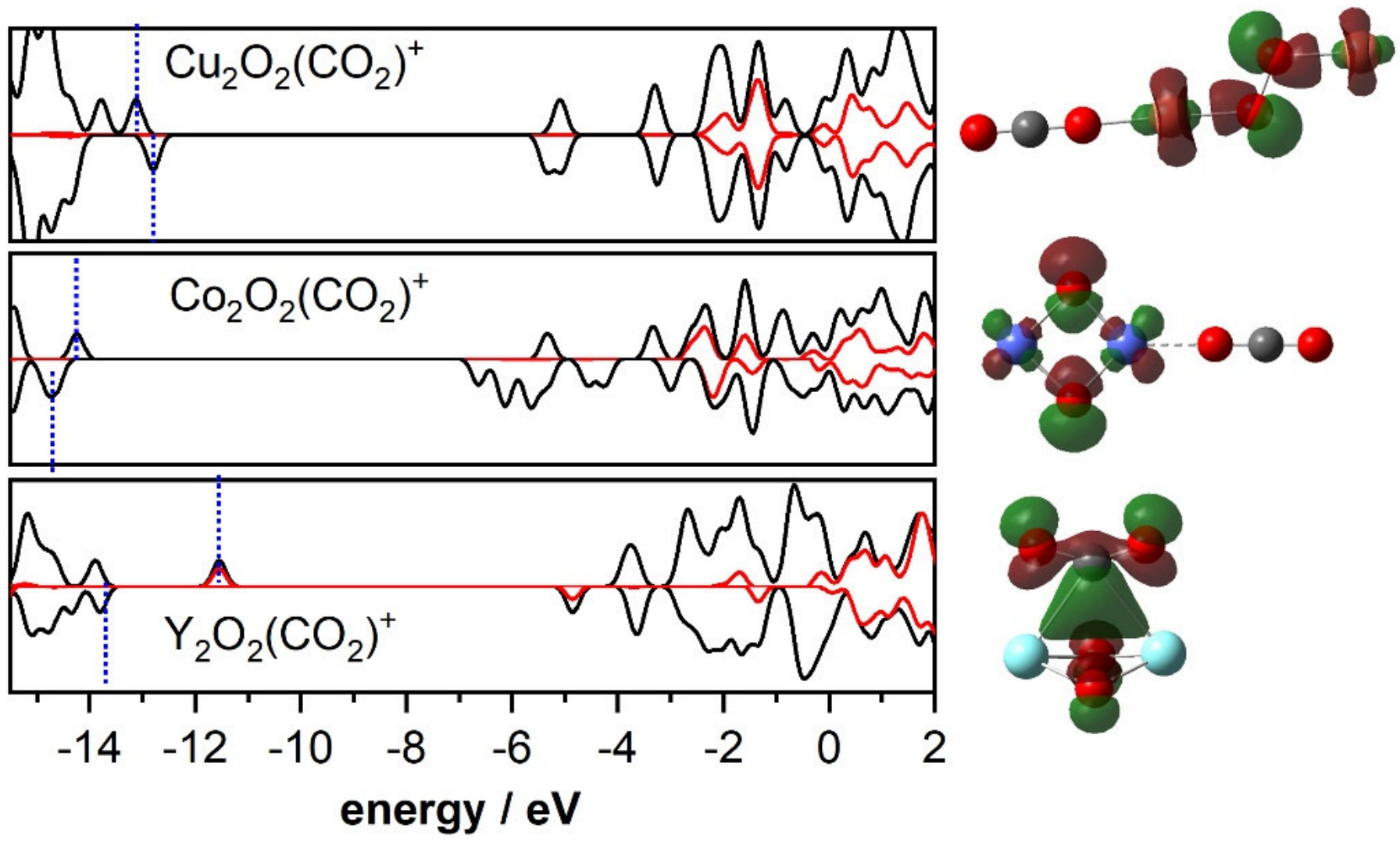

Figure S4: Total DOS (black line) of the calculated lowest energy structures of $Cu_2O_2(CO_2)^+$, $Y_2O_2(CO_2)^+$, and $Co_2O_2(CO_2)^+$ shown in Figure 6 of the main text as well as DOS projected on the CO_2 molecule (red line). The dashed blue lines indicate the α-spin and β-spin HOMOs. Also shown are the corresponding optimized geometries together with the isosurfaces (isovalue 0.05) of the HOMO.

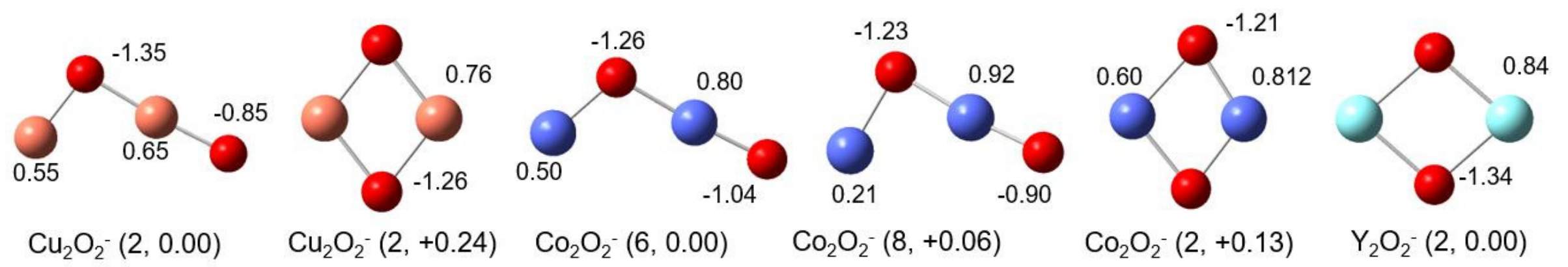

Figure S5: Optimized structure of $Cu_2O_2^-$, $Co_2O_2^-$, and $Y_2O_2^-$. The spin multiplicity and relative energy (in eV) are given in brackets. Local NBO charges (in e) are shown next to the corresponding atom.

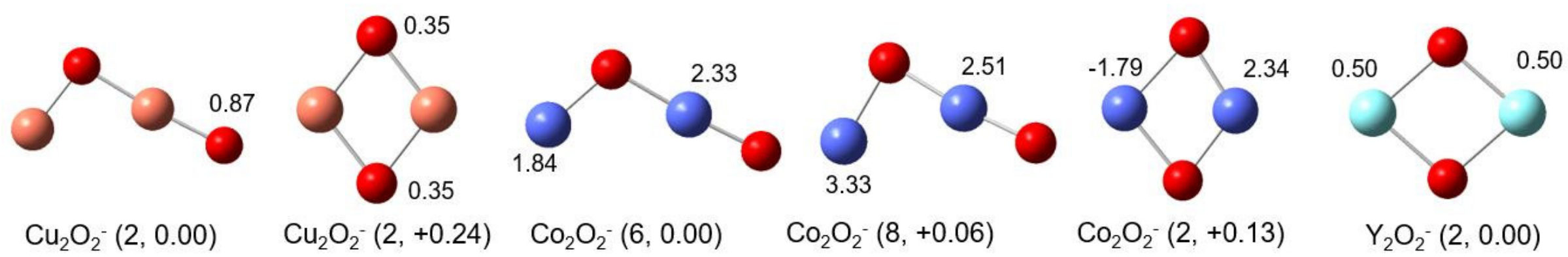

Figure S6: Optimized structure of $Cu_2O_2^-$, $Co_2O_2^-$, and $Y_2O_2^-$. The spin multiplicity and relative energy (in eV) are given in brackets. The numbers denote the major components of the excess spin.

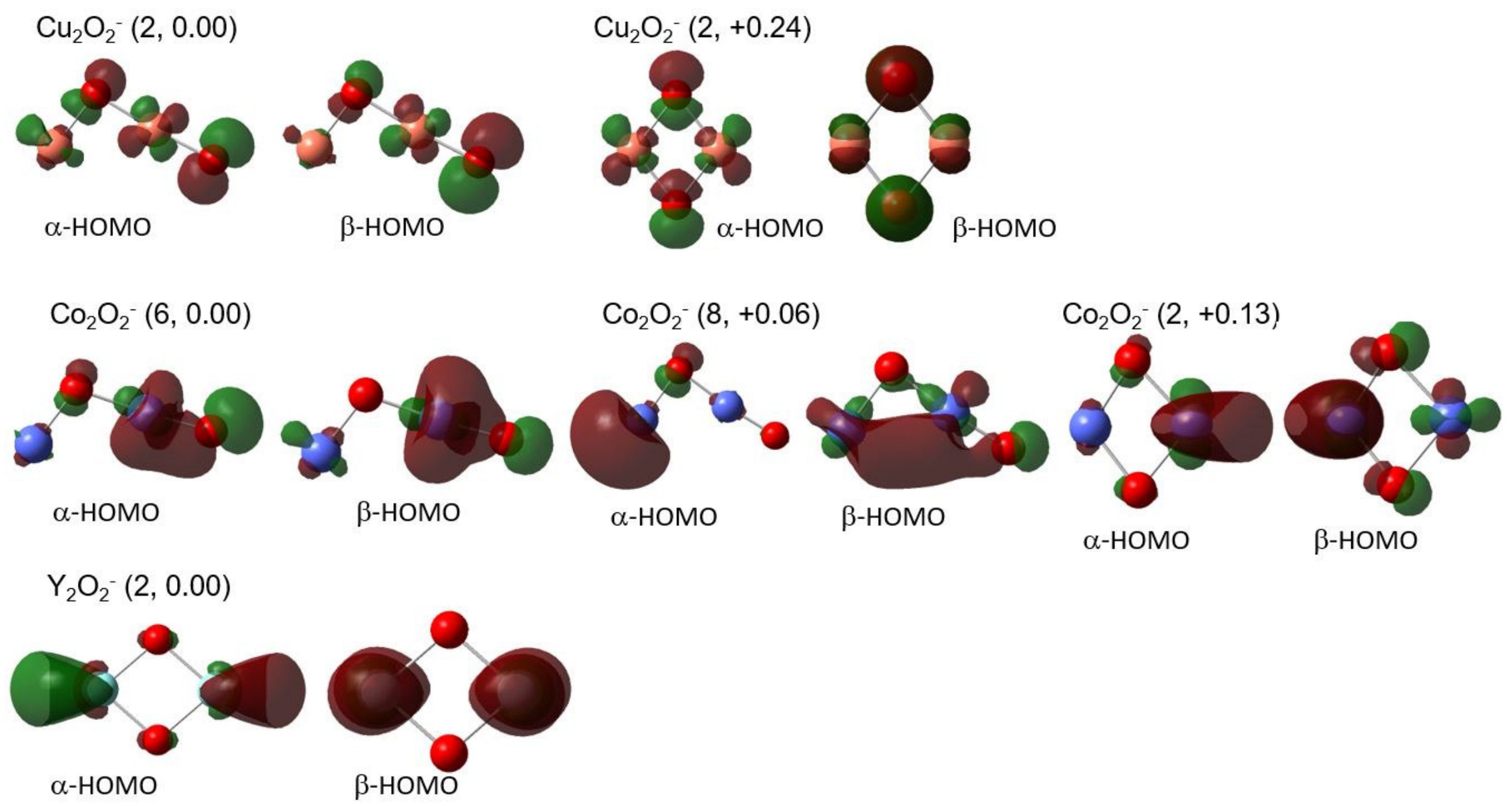

Figure S7: Optimized structures together with the isosurfaces (isovalue 0.05; except of $Y_2O_2^-$ 0.04) of the α- and β-HOMOs of $Cu_2O_2^-$, $Co_2O_2^-$ and $Y_2O_2^-$. The spin multiplicity and relative energy (in eV) are given in brackets.

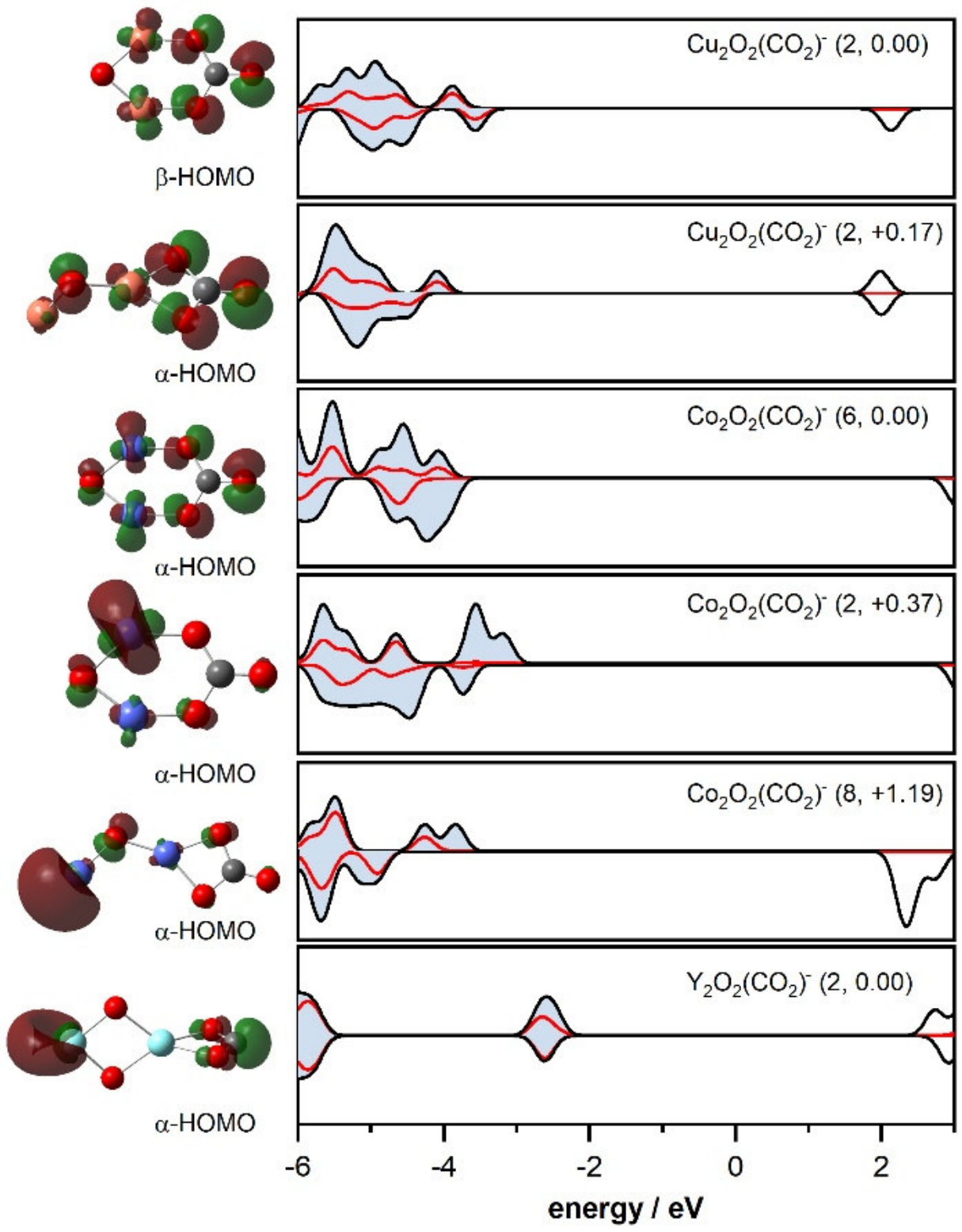

Figure S8: Total DOS (black line) of the optimized structures of $Cu_2O_2(CO_2)^-$, $Y_2O_2(CO_2)^-$, and $Co_2O_2(CO_2)^-$ as well as DOS projected on the CO_2 molecule (red line). Gray areas indicate occupied orbitals. Also shown are the corresponding optimized geometries together with the isosurfaces (isovalue 0.05) of the HOMO.

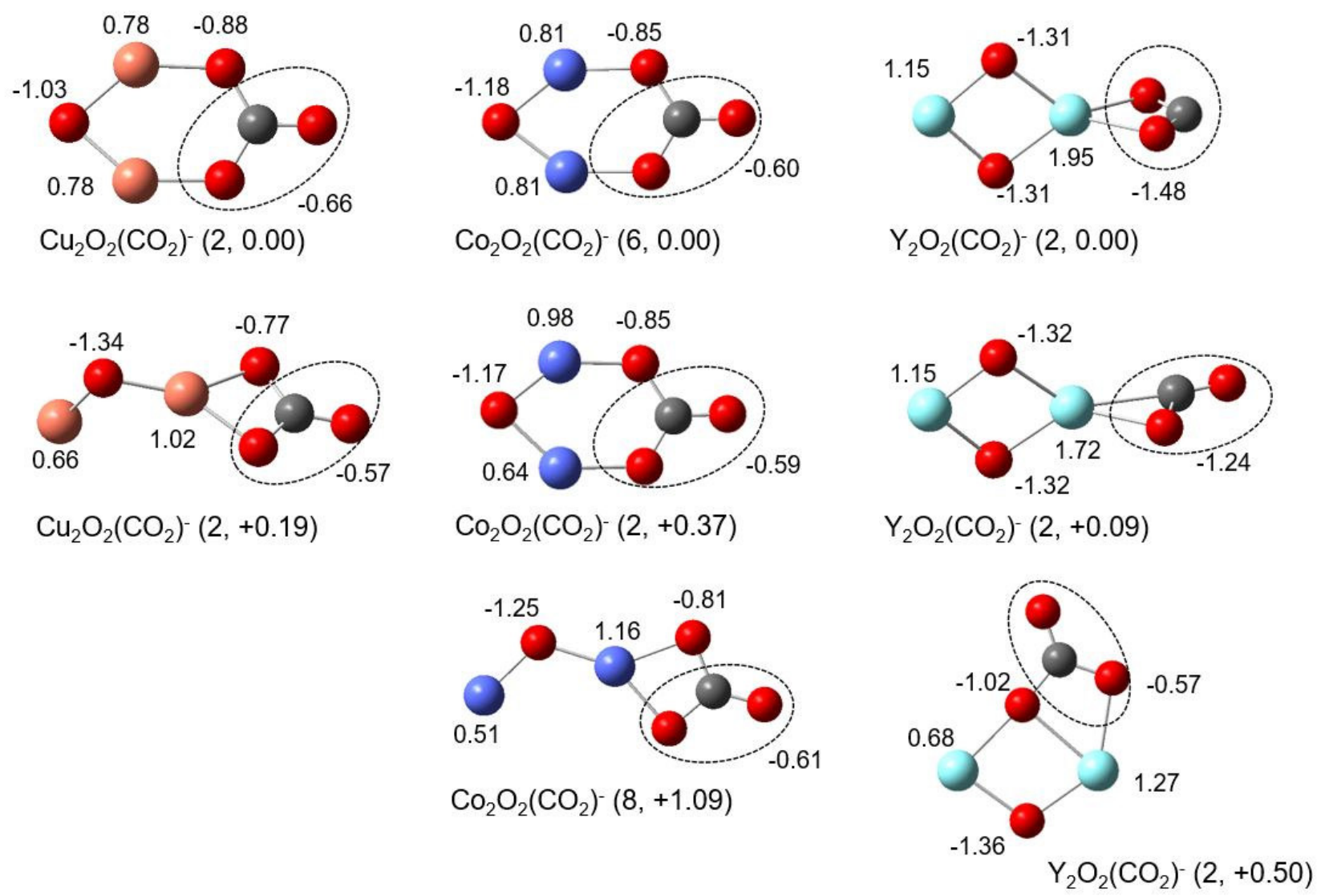

Figure S9: Optimized geometries of anionic $Cu_2O_2(CO_2)^-$, $Co_2O_2(CO_2)^-$, and $Y_2O_2(CO_2)^-$. The spin multiplicity and relative energy (in eV) are given in brackets. Local NBO charges (in e) are shown next to the corresponding atom and the adsorbed CO_2 molecule, respectively.

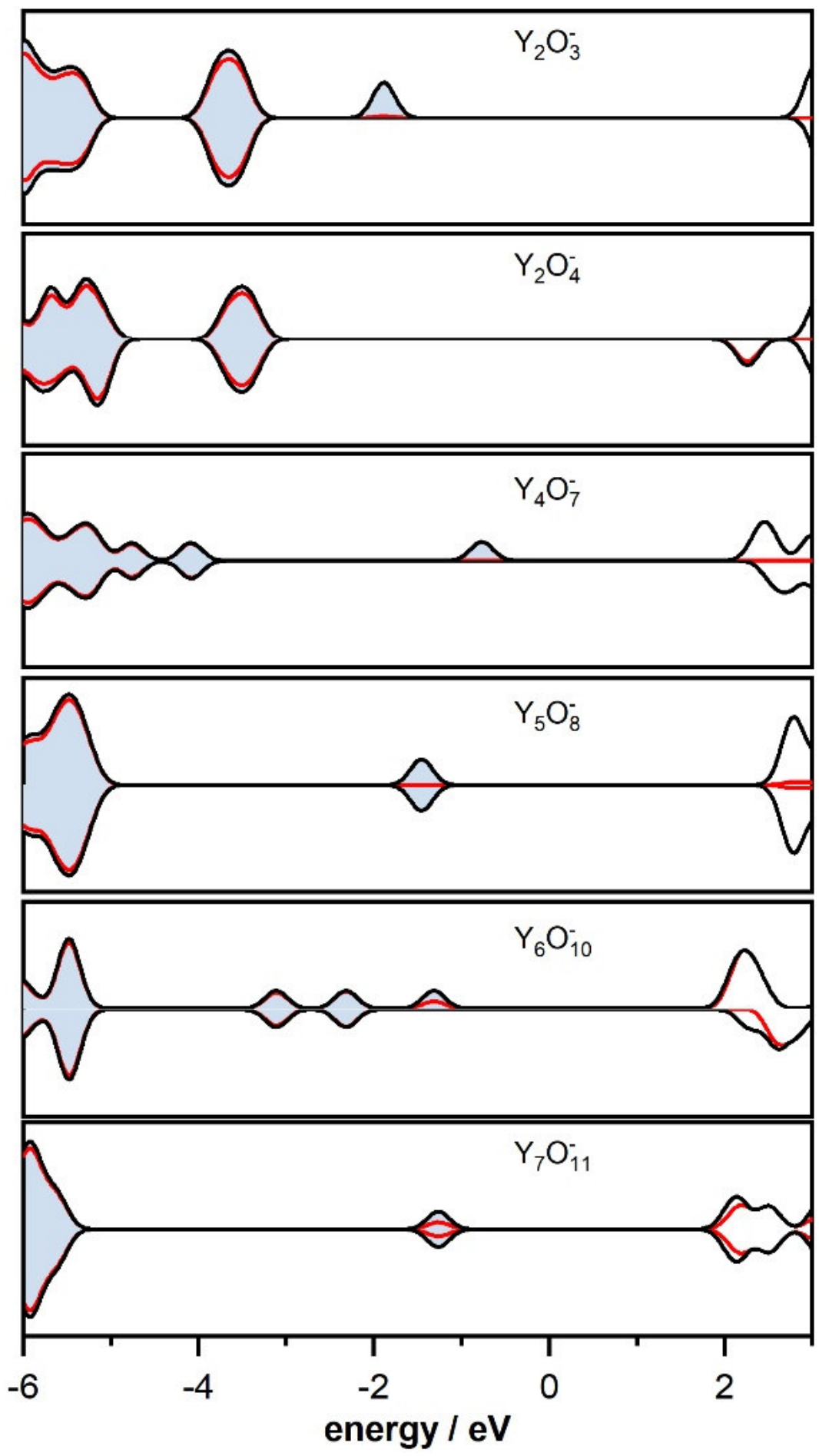

Figure S10: Total DOS (black line) of the calculated structures of Y_xO_y as well as DOS projected on the O atoms (red line). Shown are the DOS of α-spin (upper panels) and β-spin (lower panels) electrons. Gray areas indicate occupied orbitals. The structures of the clusters are shown in Figure S11.

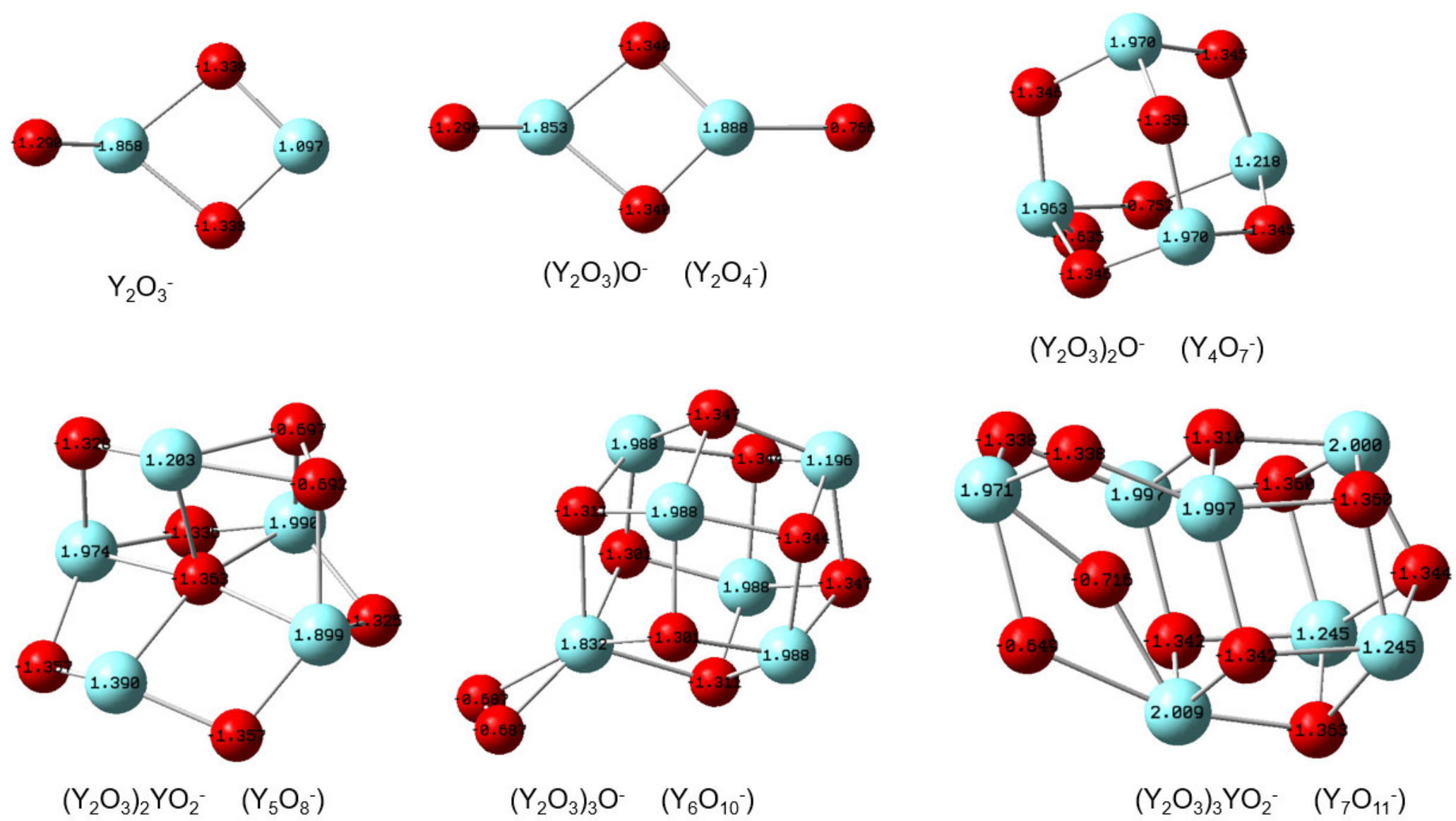

Figure S11: Optimized structure of different $Y_xO_y^-$ clusters. The structures were re-optimized based on the previously reported structures for cationic species.[2] Local NBO charges (in e) are shown on the corresponding atom.

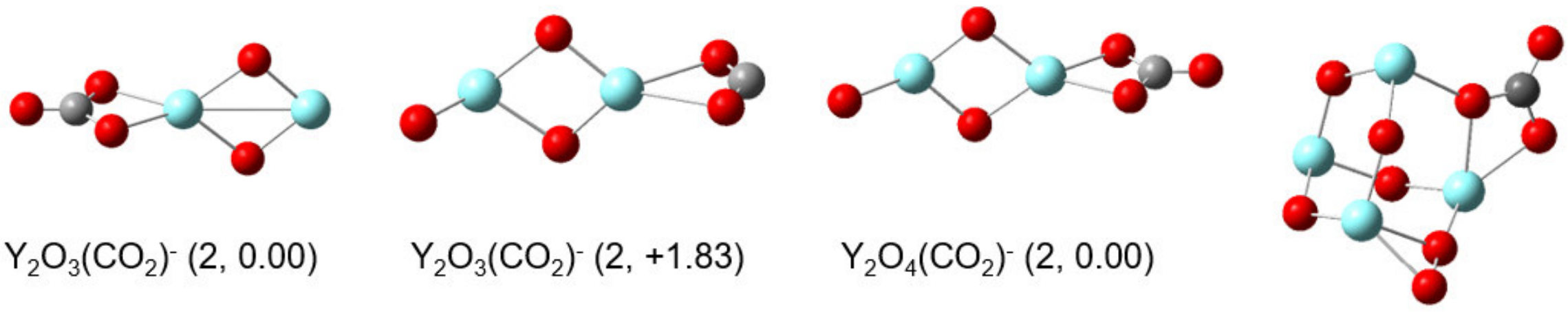

$Y_2O_3(CO_2)^-$ (2, 0.00) $Y_2O_3(CO_2)^-$ (2, +1.83) $Y_2O_4(CO_2)^-$ (2, 0.00) $Y_4O_7(CO_2)^-$ (2, 0.00)

Figure S12: Optimized structure of $Y_2O_3(CO_2)^-$, $Y_2O_4(CO_2)^-$, and $Y_4O_7(CO_2)^-$. The spin multiplicity and relative energy (in eV) are given in brackets.

References:

1. P. Mikolaj, B. Z. Yusti, L. Nyulászi, J. M. Bakker, T. Höltzl and S. M. Lang, *Phys. Chem. Chem. Phys.*, 2024, **26**, 24126-24134.
2. Y. Misao, T. Nagata, M. Nakano, K. Ohshimo and F. Misaizu, *Phys. Chem. Chem. Phys.*, 2022, **24**, 11096-11103.
